# Predicting Call Abandonment in a Health Care Call Center Using Nonpersonal Operational Data: Machine Learning Study

**DOI:** 10.2196/88441

**Published:** 2026-08-31

**Authors:** Hilmi Al-Shakhshir, Sara Kier, Dan Hoke, Stephanie Hoiriis, Mary-Ellen Vicinus, Ravi Thadhani, Anant Madabhushi

**Affiliations:** 1 Atlanta VA Medical Center Decatur, GA United States; 2 Executive Consulting Group Management Consultants Atlanta, GA United States; 3 Emory Healthcare Atlanta, GA United States; 4 Disruptive Innovations Atlanta, GA United States; 5 Department of Biomedical Engineering Wallace H. Coulter Emory University Atlanta, GA United States

**Keywords:** call abandonment, call center, CatBoost, class imbalance, health care call center, machine learning, operational analytics, patient access, random forest, SHAP, temporal validation

## Abstract

**Background:**

Call abandonment is a critical barrier to patient access in health care call centers; yet, predictive modeling efforts are limited by strict privacy regulations that restrict the use of personal or behavioral data. Whether abandonment can be accurately predicted using only anonymized operational metrics remains unclear.

**Objective:**

This study evaluated the feasibility, performance, and operational use of machine learning models trained exclusively on nonpersonal, routinely collected call center metrics to predict call abandonment across distinct organizational phases.

**Methods:**

We analyzed 1,037,363 call records from a large academic health care system spanning 4 operational periods marked by workflow changes and skill consolidation. Features included temporal variables, skill identifiers, and rolling operational metrics (in-queue time, occupancy, handle time, after-call work time, and active agents). Random forest and CatBoost models were trained on 3 phases defined as: (T1 [January-April 2023; original workflows], T2 [May-August 2023; post skill consolidation, cross-training, and new workflows], and T3a [September-December 2023; optimized processes]) using 5-fold cross-validation with 3 imbalance-handling strategies (none, class weighting, and synthetic minority oversampling technique). Temporal generalizability was assessed by evaluating all models on all 4 phases, with T3b serving as an unseen holdout set. Performance was evaluated using area under the curve (AUC), precision-recall area under the curve (PR-AUC), Brier score, and calibration error. Shapley additive explanations (SHAP) values quantified feature contributions.

**Results:**

Across all training phases and algorithms, adding operational metrics improved the area under the receiver operating characteristic curve by 0.03-0.13 vs models using only temporal and skill features. The best configuration was a CatBoost model trained on T2 with operational metrics and no imbalance correction (T3b AUC 0.767, PR-AUC 0.068, expected calibration error 0.006, and Brier 0.027). Models trained solely on preintervention data (T1) generalized poorly to postintervention periods when restricted to temporal and skill features (AUC 0.32-0.38) but achieved AUC 0.715 on T3b when operational metrics were included. SHAP analysis consistently identified in-queue time as the dominant predictor, with the number of logged-in agents, hour-of-day, and skill identifiers comprising the remaining top features. Abandonment declined from 8.7% (20,809/238,722) in T1 to 2.8% (8293/300,060) in T3b; model-based analyses of temporal features (day of week and hour of day) showed the highest risk on Mondays and between 11:00 and 16:00. Skill-level analyses showed marked improvement in high-volume imaging teams with high abandonment.

**Conclusions:**

Models trained on nonpersonal operational data predicted call abandonment on the T3b temporal holdout, with real-time queue and staffing metrics providing the dominant signal. These findings support the feasibility of interpretable, operationally grounded abandonment prediction in health care call centers and indicate that models should be retrained after major operational changes.

## Introduction

Call abandonment is when a caller drops the call while waiting to be connected to an agent. In health care call centers, call abandonment represents a critical challenge, directly affecting patient access to care, institutional revenue, and operational efficiency [1]. Health care call centers manage millions of interactions annually, with abandonment rates varying significantly across organizations, often ranging from 5% to over 20% during peak operational hours, translating into substantial revenue loss and diminished patient satisfaction [2,3]. These statistics underscore the necessity for robust strategies to mitigate call abandonment, particularly in environments where timely access to care is paramount.

Efficient call center operations are critical as they serve as the primary communication link between patients and health care providers, ensuring timely medical services and patient satisfaction [4]. Call abandonment, as a key performance metric, directly impacts operational efficiency and institutional financial health [5]. Health care call centers face unique challenges compared to other industries, including higher abandonment rates compared to retail or banking sectors, and delays in response times, leading to patient dissatisfaction, missed appointments, and reduced trust in health care systems [2,3].

Additionally, stringent privacy regulations limit the availability of the granular caller-specific data necessary for individualized predictive modeling [6,7]. Traditional predictive analytics approaches, which rely heavily on detailed personal datasets such as caller demographics and behavioral patterns, present risks to patient confidentiality and compliance with privacy standards such as the Health Insurance Portability and Accountability Act (HIPAA) [6,7]. This complicates efforts to develop effective predictive models.

Previous studies have used machine learning and queueing theory to forecast call volumes and optimize staffing. For instance, Zeltyn and Mandelbaum [1] modeled call center operations using queueing theory, while Jouini et al [8] applied stochastic models to address performance uncertainties. However, these methods often depend on sensitive data, limiting their feasibility in health care. Moreover, few studies have addressed the adaptability of predictive models to organizational transitions or fluctuating operational conditions [9].

This study addresses these gaps by exploring the feasibility of predicting call abandonment using exclusively anonymized operational metrics routinely collected in call centers, such as call date, time, requested skill, and in-queue time. By leveraging these nonpersonal operational data, our approach aims to provide actionable insights to enhance managerial decision-making, optimize resource allocation, and improve patient experiences without compromising privacy standards [1,5,10].

To address these questions, our research evaluated the feasibility of predicting call abandonment within a health care call center environment through 3 experiments, each designed to assess distinct aspects of predictive use and operational applicability. In the first experiment, we evaluated model generalizability across different operational phases. In the second, we assessed model robustness and adaptability to evolving operational conditions. Finally, the third experiment explicitly investigated the operational use of predictive analytics by examining how accurately identifying key drivers of call abandonment could inform targeted interventions at the skill-specific level. Collectively, these analyses leveraged a dataset comprising over a million calls, segmented into distinct time periods representing preintervention, transitional, and stabilized operational phases, to validate the strategic value of predictive models in health care operations management.

## Methods

### Datasets

The dataset, provided by Emory Healthcare’s call center, comprised anonymized, nonpersonal call data spanning January 2023 to May 2024, totaling over 1.2 million records from approximately 734,000 unique callers. Each call record included the contact identifier, date and time, queue duration, skill group (eg, PA Radiology Surgery and PA Imaging Echo Campaign), agent identifier, team identifier, and outcome (abandoned or connected). To comply with HIPAA, caller phone numbers were anonymized and retained only to identify repeat callers within a 24-hour window for accurate abandonment classification.

The call center system does not provide estimated wait time (EWT) announcements to callers; callers hear a standard hold message without any indication of expected wait duration. Therefore, the predictive power of in-queue time reflects system congestion conditions rather than caller-side information about anticipated waits.

The dataset was partitioned into 4 temporal phases representing distinct operational periods: T1 (preintervention: January-April 2023), T2 (transitional: May-August 2023), T3a (early stabilization: September-December 2023), and T3b (fully stabilized: January-May 2024). These phases corresponded to major organizational changes including skill consolidation, cross-training initiatives, and workflow optimization. T3b served as an unbiased holdout set, never used for model training, enabling evaluation of temporal generalizability.

### Data Preparation and Feature Engineering

Data cleaning was systematically performed using the R *tidyverse* package [11] to ensure dataset integrity. Records missing essential attributes (queue duration, outcome, or time stamp) were excluded. Call abandonment was defined as a caller-initiated disconnection before agent connection, following the standard industry definition where any disconnect prior to service constitutes an abandonment. This excludes technical disconnections (system drops logged separately in the automatic call distributor system) and interqueue transfers, which maintain call connections.

To assess label noise robustness, we conducted a false abandonment sensitivity analysis identifying callers who abandoned but successfully reconnected within 1-hour, 4-hour, 12-hour, and 24-hour windows using the anonymized caller identifier (master_contact_id). False abandonments represented only 1.2%-1.7% of abandoned calls across all phases, and all reconnections occurred within the first hour. Excluding these calls changed model area under the receiver operating characteristic curve (ROC-AUC) by only +0.003 (Table S2 and Figure S12 in Multimedia Appendix 1), confirming that the primary results are robust to this label noise.

### Skill Canonicalization

To maintain consistency across operational phases, historical skill labels were mapped to the current taxonomy for a defined subset of legacy queues. A total of 12 legacy skills were consolidated into 3 primary skill groups via exact-match recoding: PA Radiology Surgery (encompassing “PA Radiology-Nutrition,” “PA Radiology-Cardiology,” “PA Radiology-Gen Surgery,” and “PA Radiology-Respiratory”), PA Radiology Team Blue Magic (consolidating “PA Radiology-CT Scan,” “PA Radiology-Cardiac PET,” “PA Radiology-Nuc Med,” and “PA Radiology-IR NR”), and PA Radiology Team Rad Achiever (combining “PA Radiology-MRI,” “PA Radiology-Ultrasound,” “PA Radiology-Gen Diagnostics,” and “PA Radiology-Infusion”). This canonicalization ensured feature consistency across all temporal phases for valid model comparison within these 3 groups.

A separate set of legacy scheduling queues, “Imaging-Mammogram Scheduling,” “Imaging-CT Scheduling,” “Imaging-MRI Scheduling,” “PA Interventional Radiology,” and “PA Radiology-IR RN,” was retained as distinct skill categories and was not folded into the consolidated groups; these queues are not part of the 12-skill consolidation and remain separately labeled throughout the dataset. Their call volumes declined substantially, in several cases to near zero, in T3a and T3b as call routing shifted away from these legacy queues; we address the implications in the Limitations section.

### Temporal Feature Encoding

Temporal features were engineered to capture cyclical patterns in call center operations. Hour of day (0-23) and day of week (Monday-Sunday) were encoded using sine and cosine transformations to preserve their cyclical nature, preventing artificial discontinuities at period boundaries. For a cyclical variable x with period p, the transformation is defined as:



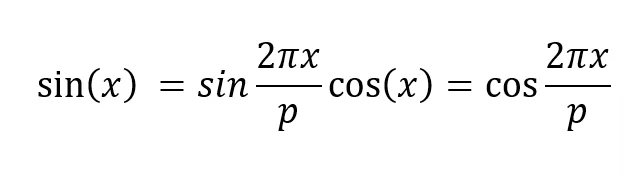



This encoding maps temporal features onto unit circles, ensuring that adjacent time points (eg, 11 PM to 12 AM) are represented as proximate in the feature space rather than numerically distant. A binary holiday indicator was derived using the US federal holiday calendar (*timeDate* package).

### Rolling Operational Metrics

To capture operational dynamics available at prediction time, we computed rolling metrics over 60-minute trailing windows within each skill, restricted to information knowable at the moment of each call’s arrival (time T). A prior call contributes its in-queue time to inqueue_roll_60m only if it had completed waiting (been answered or abandoned) by T; its handle time to aht_roll_60m only if handling had completed by T; and its after-call work time to acw_roll_60m only if wrap-up had completed by T; agents_logged_in counts distinct agents observed answering calls within the window by T, and occupancy_proxy divides completed busy time by that staffed capacity (defined as 0 when no capacity had been observed). The current call is always excluded from its own windows. Calls with no eligible contributors (eg, the first call after a quiet period) receive missing values; these are median-imputed within the modeling pipeline with accompanying missingness-indicator variables, so that data-sparse periods are represented explicitly rather than as typical load.

### Model Development

#### Algorithms and Feature Sets

Two machine learning algorithms were evaluated: random forest [12] with 800 trees and CatBoost [13] (gradient boosting on decision trees). Each algorithm was trained using 2 feature configurations: BASE (skill group, temporal features with cyclic encoding, holiday indicator) and WITH_AGENTS (BASE features plus rolling operational metrics).

#### Imbalance Handling Strategies

Three approaches to address class imbalance were systematically compared [14,15]: (1) None (baseline), (2) class weighting (inverse frequency weighting: *w_minority_=n_total_*/(2×*n_minority_*), *w_majority_=n_total_*/(2×*n_majority_*), and (3) synthetic minority oversampling technique (SMOTE) with over_ratio=0.25. Class weighting adjusts the loss function to penalize misclassifications proportionally to class rarity, while SMOTE generates synthetic minority class examples through k-nearest neighbors interpolation (k=5, adaptively adjusted based on minority class size per fold).

#### Training Protocol

Models were trained independently on each phase (T1, T2, and T3a) using 5-fold stratified cross-validation (CV) with the outcome (abandon: yes/no) as the stratification variable. We implemented preprocessing pipelines, using the *recipes* R package, that included factor encoding, imputation (median for numeric and mode for categorical), and SMOTE augmentation where applicable. SMOTE augmentation was strictly isolated to training data within each CV fold. Training data was extracted via juice (recipe_prep), which returns the augmented training set including synthetic samples. Validation and test data were processed via bake(recipe_prep, new_data), which applies only deterministic transformations (encoding, imputation) and never generates synthetic observations. No synthetic samples contaminated validation folds or the T3b holdout set. All models within each algorithm-feature-variant configuration used consistent hyperparameters to isolate the effects of training phase, feature selection, and imbalance handling. To verify that performance differences reflected algorithmic architecture rather than default parameter suitability, we subsequently performed systematic hyperparameter optimization using nested 3-fold CV within each training phase. For random forest, we searched over mtry (2-8) and min_n (5, 10, 20, 50); for CatBoost, we searched over depth (4, 6, 8, 10) and learning_rate (0.01, 0.05, 0.10). Tuned models were evaluated on the T3b holdout set (Figures S13a-S13c and Table S3 in Multimedia Appendix 1).

Each combination of 3 training phases, 2 algorithms, 2 feature sets, and 3 imbalance-handling methods yielded 36 trained models, with full refit on entire phase data post CV for subsequent temporal validation.

#### Baseline Models

To quantify the marginal use of ensemble methods, we implemented 4 baseline models evaluated on the same T3b holdout set: (1) logistic regression with L2 regularization using the same with_agents feature set; (2) an Erlang-A queuing model (M/M/s+M) parameterized using phase-specific arrival rates, service rates, and exponentially modeled caller patience estimated by maximum likelihood under right-censoring; (3) a temporal heuristic predicting abandonment probability from day-of-week and hour-of-day historical rates alone; and (4) a prevalence-only baseline predicting the marginal abandonment rate for all calls.

#### Temporal Validation Strategy

Following established practices for time series evaluation [16,17], each trained model was tested on all 4 temporal phases (T1, T2, T3a, and T3b) to assess generalizability and temporal stability. This design does not strictly preserve temporal ordering, because some models were evaluated on data from earlier phases than the period used for training; these results were used descriptively to compare performance across operational regimes. To mimic a prospective deployment scenario, we treated the final phase (T3b) as a future holdout set and emphasized model performance on this period.

The preprocessing pipeline (feature encoding, imputation, and skill mapping) from training was applied to each test set without refitting, maintaining realistic prediction conditions. This yielded 144 model-test combinations (36 trained models × 4 test phases), enabling comprehensive assessment of (1) within-phase performance (model trained and tested on same phase), (2) forward generalization (model tested on later phases), and (3) cross-phase robustness (model tested across disparate operational contexts).

#### Blocked Temporal Cross-Validation

To assess sensitivity to temporal autocorrelation, we implemented expanding-window blocked temporal CV alongside standard 5-fold CV. Each training phase (~4 months) was split into monthly blocks, and we used an expanding-window protocol: train on month 1, test on month 2; train on months 1-2, test on month 3; train on months 1-2-3, test on month 4. This protocol respects the temporal ordering of observations and reduces potential leakage from short-term operational patterns.

#### Performance Metrics

Model discrimination was evaluated using the ROC-AUC with DeLong 95% CIs and precision-recall area under the curve (PR-AUC) with bootstrap CIs (500 resamples). Calibration quality was assessed via Brier score (mean squared error between predicted probabilities and binary outcomes), and expected calibration error (ECE) computed across 10 probability bins as the weighted average of absolute differences between bin accuracy and mean confidence.

For clinical interpretability, models were also evaluated at specific operating thresholds using sensitivity (recall), specificity, and *F*_1_-score (harmonic mean of precision and recall). These metrics provided complementary perspectives on model performance relevant to different stakeholder priorities. Overall classification accuracy was not used as a performance metric due to the severe class imbalance (2.8% abandonment prevalence in the holdout set). A naive classifier predicting all calls as nonabandoned would achieve 97.2% accuracy, making this metric uninformative for evaluating model discrimination.

#### Threshold Optimization and Cost-Benefit Analysis

Optimal decision thresholds were identified through multiple criteria: (1) *F*_1_-score maximization, (2) Youden index (sensitivity + specificity – 1), and (3) cost minimization. A simple cost model assigned a unit cost to false positives (FPs; unnecessary intervention) and a 5-fold cost to false negatives (FNs; missed abandonments). An extended time-varying cost model incorporated differential costs for peak hours (9:00-17:00: FP cost=1.5; FN cost=6.0) vs off-peak hours (FP cost=0.7; FN cost=4.0), reflecting varying operational constraints and patient impact across the day.

For each model-test combination, thresholds were swept from 0.01 to 0.99 in 0.02 increments, computing performance metrics at each point. Bootstrap CIs (50 resamples) quantified threshold stability for groups exceeding 5000 calls. Expected cost reduction and intervention volume were estimated for each policy to inform operational planning. To assess sensitivity to cost assumptions, we conducted a cost ratio sweep varying the FN:FP ratio from 1:1 to 30:1, examining how the optimal decision threshold and operating characteristics change under different assumptions about the relative cost of missed abandonments vs unnecessary interventions (Figure S8 in Multimedia Appendix 1).

#### Model Interpretability

Shapley additive explanations (SHAP) [18,19] values were computed to quantify feature contributions to predictions, providing model-agnostic interpretability. For each trained model, SHAP values were calculated on a stratified sample of 10,000 calls per test phase using 25 Monte Carlo simulations. Feature importance was aggregated as the mean absolute SHAP value across observations, and directional effects were visualized through SHAP dependence plots. This analysis identified which operational factors (eg, in-queue time, hour of day, and specific skills) most strongly influenced abandonment predictions and whether their effects remained consistent across temporal phases.

#### Statistical Analysis

Point-biserial correlation assessed the association between queue duration (continuous) and abandonment (binary), both globally and within each phase, quantifying the strength of this established relationship and detecting temporal variations. Chi-square tests identified significant differences in abandonment rates across categorical predictors (skill group, hour, and month), highlighting operational vulnerabilities through comparison of observed vs expected frequencies.

General linear models examined call volume patterns while controlling for skill, hour, and month, testing for interaction effects and identifying peak demand periods requiring targeted resource allocation. Model comparison used comprehensive ranking across ROC-AUC, PR-AUC, Brier score, and ECE, with primary evaluation on the T3b holdout set. All analyses were conducted using R software (version 4.3.1; R Foundation for Statistical Computing) with packages including *tidyverse* [11], *ranger* (random forest implementation) [20], *catboost* (gradient boosting) [13], recipes/workflows (preprocessing and modeling framework), *fastshap* (SHAP computation) [19], *pROC* (receiver operating characteristic analysis) [21], and *PRROC* (precision-recall curves).

### Ethical Considerations

This study did not require institutional review board (IRB) review or approval. In accordance with Emory University’s documented institutional determination process (Emory IRB memorandum of July 11, 2019, “Documentation for projects that do not require IRB review”), the project was evaluated using the Emory IRB Non-Human Subjects Research Determination electronic form (completed July 14, 2026), with the recorded determination that the project is not research with human participants, nor a clinical investigation, as defined under the US Department of Health and Human Services regulations at 45 CFR 46.102(e) [22], because the analysis uses exclusively deidentified operational call center data (call time stamps, queue metrics, staffing levels, and call disposition). The completed determination form is retained as institutional documentation and is available on request.

Informed consent was not applicable, as no human participants were involved and only retrospective operational records were analyzed. The dataset contains no patient names, medical record numbers, phone numbers, or other personally identifiable information; caller identifiers (master_contact_id) are system-generated hashes that cannot be linked back to individuals. No participants were enrolled or compensated. No images in the manuscript or supplementary materials contain identifiable individuals.

## Results

### Dataset Characteristics and Operational Context

**Figure 1:**
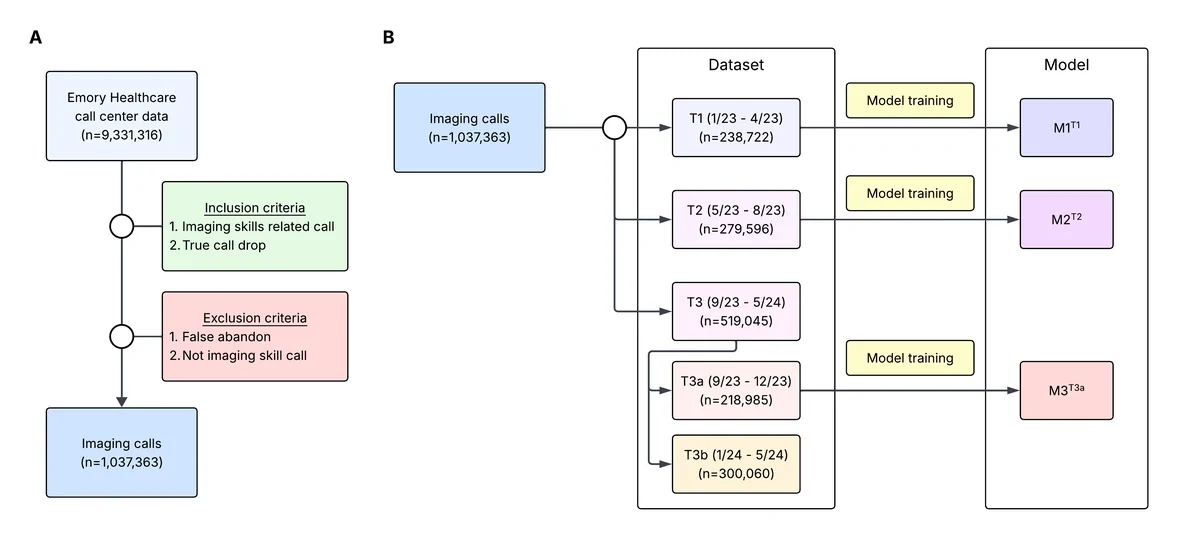
CONSORT (Consolidated Standards of Reporting Trials) chart and study design. (A) The CONSORT flowchart illustrating data flow from the initial dataset through inclusion and exclusion criteria to final analysis set. The initial Emory Healthcare Call Center dataset (n=9,331,316 calls) was filtered using inclusion criteria (imaging skills-related calls and true call drops) and exclusion criteria (false abandons and nonimaging skill calls), resulting in the final imaging calls dataset (n=1,037,363). (B) The study design showing dataset partitioning and model training structure. The imaging calls were divided into 4 temporal phases: T1 (preintervention: January-April 2023, n=238,722), T2 (transitional: May-August 2023, n=279,596), T3 (September 2023-May 2024, n=519,045), subdivided into T3a (early stabilization: September-December 2023, n=218,985) and T3b (holdout validation: January-May 2024, n=300,060). Three models were trained: M1 (superscript T1) on the T1 dataset, M2 (superscript T2) on the T2 dataset, and M3 (superscript T3a) on the T3a dataset, with T3b reserved as an independent holdout set for temporal validation.

**Figure 2:**
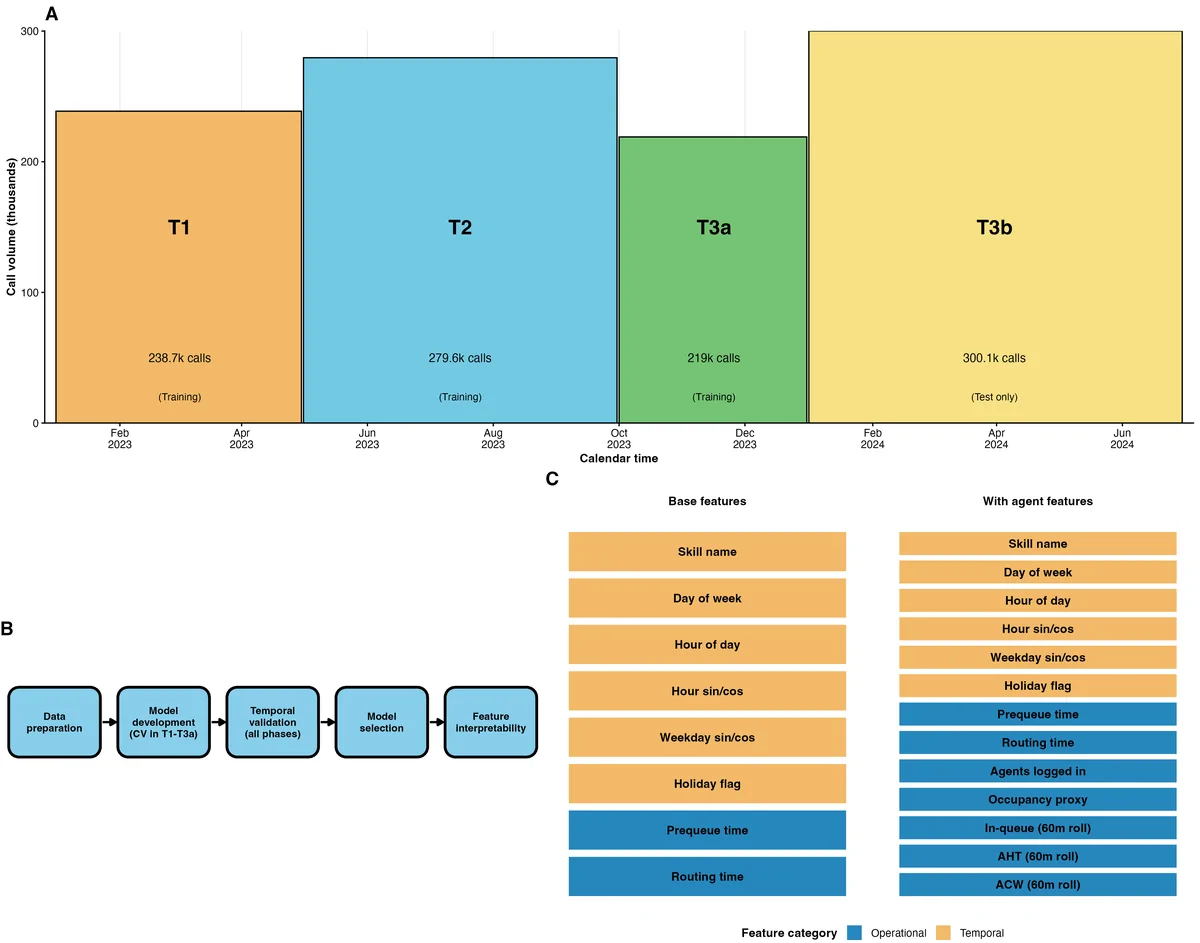
Study design and analytical framework overview of study design, temporal phases, and analytical approach for call abandonment prediction. (A) Temporal study phases with call volumes across 4 operational periods (T1: preintervention, T2: transitional, T3a: early stabilization, T3b: holdout validation). (B) The analytical workflow from data processing through model development, temporal validation, and interpretability analysis. (C) Comparison of feature sets, contrasting base features (temporal patterns and skill assignments) with operational features (including agent metrics, queue times, and occupancy rates). The dataset comprised 1,037,363 call records spanning 17 months across organizational transitions involving skill consolidation, cross-training, and workflow optimization. ACW: after-call work; AHT: average handle time; CV: cross-validation.

The final analytic dataset included 1,037,363 call records spanning January 2023 to May 2024, corresponding to 4 operational phases: T1, T2, T3a, and T3b (Figures 1 and 2; Figure S1 in Multimedia Appendix 1). Abandonment rates declined from 8.7% (20,809/238,722) in T1 to 2.8% (8293/300,060) in T3b (Table 1). Mean in-queue time decreased from 279 seconds in T1 to approximately 50 seconds in T3a and T3b, and median queue times decreased from 21-25 seconds to 2 seconds. Staffing levels during business hours ranged from 14-17 active agents, and mean occupancy under the corrected (knowability-gated) definition ranged between 31% (SD 14.1%) and 42% (SD 22.5%; Table 1). A sensitivity analysis confirmed that false abandonments (callers who abandoned but reconnected within 24 hours) represented only 1.2%-1.7% of abandoned calls and had negligible impact on model performance (ROC-AUC delta: +0.003; Figure S12 in Multimedia Appendix 1).

**Table 1:** Operational characteristics by phase: summary of operational metrics across 4 temporal phases (T1: preintervention, T2: transitional, T3a: early stabilization, and T3b: holdout validation). Metrics include total calls, abandoned calls, abandonment rate, mean and median in-queue time, average handle time (AHT), after-call work (ACW) time, mean agents logged in, and occupancy proxy. The table demonstrates substantial improvement in abandonment rates from 8.7% in T1 to 2.8% in T3b, accompanied by reductions in queue times while maintaining stable staffing levels.

Phase	Total calls	Abandoned calls, n (%)	In-queue time (seconds), mean (SD)	In-queue time (seconds), median (IQR)	AHT 60 m rolling (seconds), mean (SD)	ACW 60 m rolling (seconds), mean (SD)	Agents logged in, mean (SD)	Occupancy (%), mean (SD)
T1	238,722	20,809 (8.7)	279.5 (553.8)	21.0 (2.0-306.0)	233.1 (72.4)	73.3 (52.5)	13.6 (5.9)	31.4 (14.1)
T2	279,596	15,122 (5.4)	132.5 (298.0)	25.0 (2.0-149.0)	240.3 (61.4)	74.8 (60.4)	15.0 (8.1)	41.9 (22.5)
T3a	218,985	5725 (2.6)	49.4 (106.1)	2.0 (2.0-56.0)	252.4 (57.3)	74.5 (54.4)	17.1 (8.0)	34.6 (11.9)
T3b	300,060	8293 (2.8)	53.4 (114.0)	2.0 (2.0-61.0)	251.4 (57.9)	78.1 (57.2)	16.3 (7.9)	35.7 (12.6)

### Model Development and Cross-Validation Performance

**Figure 3:**
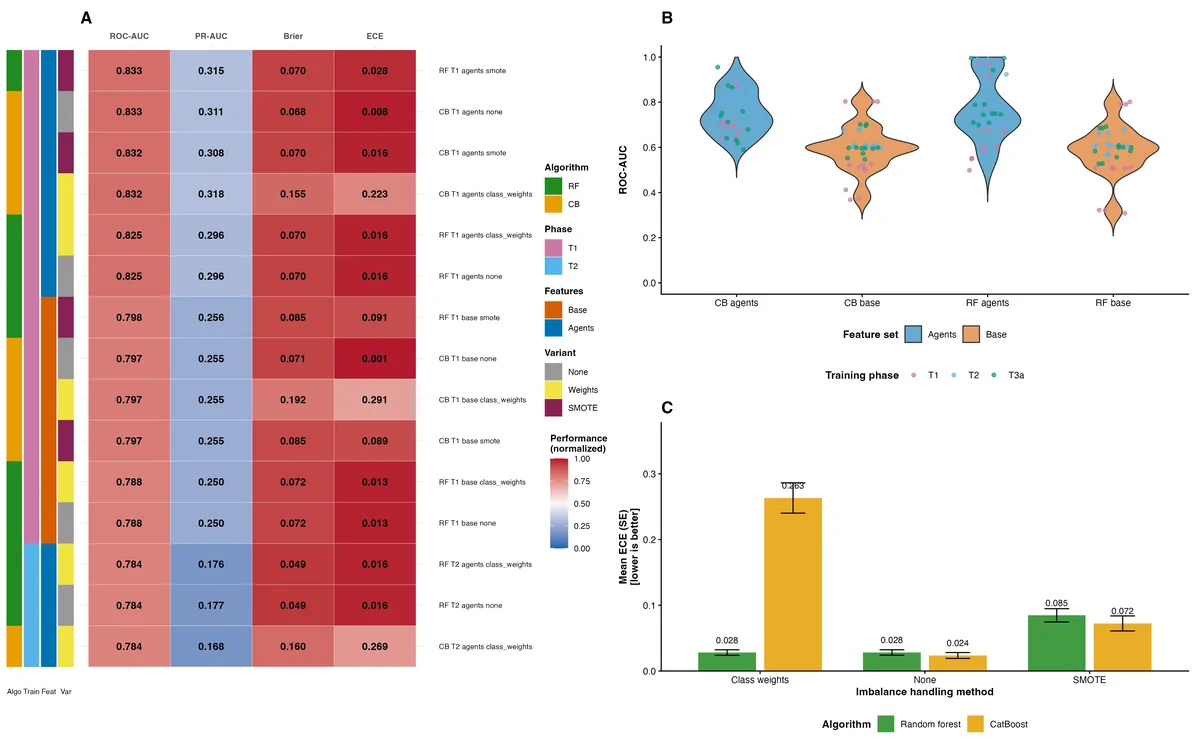
Model performance comparison across configurations: systematic comparison of model performance across algorithms, training phases, feature sets, and imbalance handling strategies. (A) A heatmap of 5-fold cross-validation performance (area under the receiver operating characteristic curve [ROC-AUC], precision-recall area under the curve [PR-AUC], Brier score, and expected calibration error [ECE]) for the top 15 of 36 model configurations ranked by cross-validated ROC-AUC, with row annotations for algorithm, training phase, feature set, and imbalance variant. (B) Violin plots of ROC-AUC across all 144 model-test-phase combinations, grouped by algorithm and feature set and colored by training phase, demonstrating the consistent improvement from operational (with_agents) features. (C) Mean ECE (SE) by imbalance handling method and algorithm, showing that models without imbalance correction achieve the best calibration and that class weighting severely degrades CatBoost calibration (mean ECE 0.263, SD 0.113) while leaving random forest (RF) calibration essentially unchanged (mean ECE 0.028, SD 0.020). Lower ECE values indicate better-calibrated probability estimates. SMOTE: synthetic minority oversampling technique.

Cross-validation results varied by training phase and feature configuration (Figure 3A; Table 2). Across all phases and algorithms, incorporating operational features (in-queue time, handle time, after-call work time, occupancy, active agent count) improved cross-validated ROC-AUC by 0.03-0.13 compared with BASE models using only skill and temporal features (smallest for T1-trained CatBoost and largest for T2-trained models); on the T3b holdout the corresponding gains were 0.11-0.34 (Table S7 in Multimedia Appendix 1).

**Table 2:** Model performance leaderboard (top 10 models on T3b holdout): ranking of top 10 model configurations by performance on the T3b holdout set. Columns include rank, training phase, algorithm, feature set, imbalance handling method, area under the curve (AUC), precision-recall AUC (PR-AUC), Brier score, and expected calibration error (ECE). The leaderboard reveals that models trained on postintervention data (T2 and T3a) with operational metrics and no imbalance correction achieved the strongest prospective performance, with the top-ranked model (CatBoost trained on T2) achieving an AUC of 0.767 and an ECE of 0.006. Aggregate algorithm-level performance is summarized in Table S10 in Multimedia Appendix 1, and the complete 36-model leaderboard is provided in Table S11 in Multimedia Appendix 1.

Rank	Training phase	Algorithm	Feature set	Variant	CV^a^ AUC (95% CI)	CV PR-AUC	CV ECE	T3b AUC (95% CI)	T3b PR-AUC	T3b ECE	T3b Brier
1	T2	CatBoost	With agents	None	0.778 (0.775-0.782)	0.166	0.002	0.767 (0.762-0.771)	0.068	0.006	0.027
2	T2	CatBoost	With agents	SMOTE^b^	0.772 (0.769-0.776)	0.161	0.021	0.763 (0.759-0.768)	0.068	0.011	0.027
3	T2	CatBoost	With agents	Class weights	0.784 (0.780-0.787)	0.168	0.269	0.761 (0.756-0.765)	0.071	0.171	0.084
4	T3a	CatBoost	With agents	None	0.757 (0.751-0.763)	0.077	0.004	0.760 (0.755-0.764)	0.070	0.007	0.026
5	T3a	CatBoost	With agents	SMOTE	0.753 (0.747-0.760)	0.076	0.016	0.752 (0.747-0.756)	0.068	0.010	0.027
6	T3a	Random forest	With agents	SMOTE	0.757 (0.751-0.763)	0.078	0.037	0.749 (0.744-0.754)	0.069	0.033	0.029
7	T3a	Random forest	With agents	None	0.750 (0.744-0.756)	0.069	0.012	0.746 (0.741-0.751)	0.068	0.007	0.027
8	T3a	Random forest	With agents	Class weights	0.750 (0.744-0.756)	0.070	0.012	0.744 (0.739-0.749)	0.068	0.007	0.027
9	T3a	CatBoost	With agents	Class weights	0.748 (0.741-0.754)	0.077	0.214	0.739 (0.734-0.744)	0.066	0.169	0.092
10	T2	Random forest	With agents	SMOTE	0.782 (0.778-0.785)	0.169	0.034	0.724 (0.719-0.730)	0.061	0.040	0.030

^a^CV: cross-validation.

^b^SMOTE: synthetic minority oversampling technique.

### Baseline Comparison

Baseline comparisons demonstrated substantial machine learning uplift on the T3b holdout. Logistic regression achieved an ROC-AUC of 0.736, confirming that operational features carry a strong linear signal. However, CatBoost achieved an absolute improvement of 0.031 ROC-AUC (3.1 percentage points; 4.2% relative improvement) over logistic regression (0.767 vs 0.736), with gains concentrated in the high-risk tail of the distribution. The Erlang-A queuing model (ROC-AUC=0.561) and temporal heuristic (ROC-AUC=0.562) performed only marginally above random, demonstrating that classical queuing theory and simple time-of-day patterns alone are insufficient for call-level abandonment prediction. The temporal heuristic’s near-random performance demonstrates that day-of-week and hour-of-day patterns alone, while useful for aggregate staffing, cannot predict individual call outcomes (Figure S9 and Table S5 in Multimedia Appendix 1).

Models trained on T1 achieved CV area under the curve (AUC) of approximately 0.83, while those trained on T2 and T3a achieved AUC values of 0.748-0.784. CatBoost models demonstrated marginally better calibration than random forest, though both showed similar feature importance rankings (Figure S5 in Multimedia Appendix 1).

Imbalance-handling strategies showed distinct calibration patterns. Models trained without imbalance correction achieved the lowest ECE (mean ECE ≤0.04 across configurations), whereas class-weighted CatBoost models showed markedly degraded calibration (mean ECE 0.13-0.35; class weighting left random forest calibration essentially unchanged) and SMOTE variants showed intermediate calibration (mean ECE 0.01-0.15; Figure 3C; Table S9 in Multimedia Appendix 1). The minority class remained substantial across phases (>5700 abandoned calls per phase). Point-biserial correlations between in-queue time and abandonment ranged from *r*=0.18-0.31.

### Temporal Validation and Generalizability

**Figure 4:**
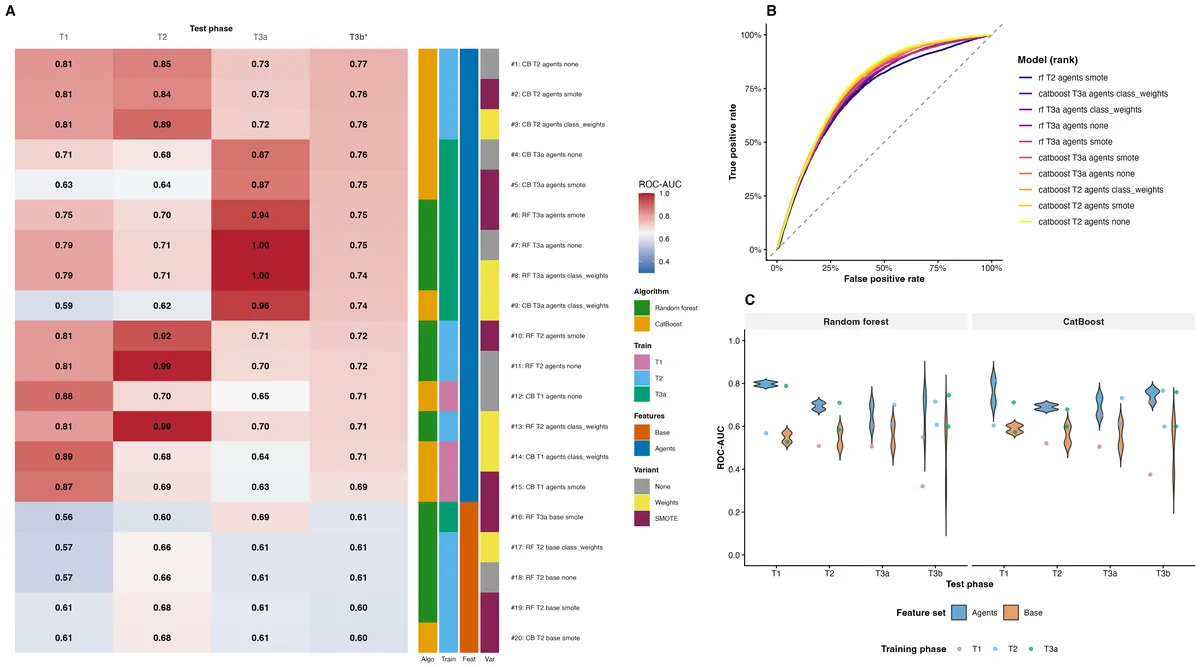
Temporal validation and generalizability: comprehensive temporal validation showing model performance across time periods and operational regimes. (A) A heatmap of the top 20 models evaluated across 4 temporal test phases (T1, T2, T3a, and T3b), with area under the curve (AUC) values shown for each model-phase combination. Diagonal (within-phase) cells display within-phase in-sample AUC, as in Table S8 in Multimedia Appendix 1, not the 5-fold cross-validation AUC reported in Table 2; the 2 metrics differ by construction. Models are ordered by T3b holdout performance and include both with_agents and base-feature variants to allow visual comparison of the contribution of operational features; the 3 base-feature configurations appearing at the bottom of the ranking (rows 18-20) achieve substantially lower discrimination across all test phases. Color intensity indicates discrimination quality, revealing that models trained on recent data (T2 and T3a) maintain consistent performance across phases, while models trained on preintervention data (T1) show marked degradation on postintervention test sets. (B) Receiver operating characteristic curves for the top 5 performing models on the T3b holdout set, with AUC values ranging from 0.752 to 0.767. (C) compares temporal stability across model characteristics, showing mean AUC and SD across test phases stratified by training phase, feature set, and algorithm. ROC-AUC: area under the receiver operating characteristic curve; SMOTE: synthetic minority oversampling technique.

Models trained on postintervention data (T2 or T3a) demonstrated the strongest forward generalization, with AUC values of 0.72-0.77 on the T3b holdout set; unweighted and SMOTE variants maintained ECE ≤0.04, while class-weighted variants showed degraded calibration (Figure 4; Table 2). The highest-performing configuration was CatBoost trained on T2 with operational metrics and no imbalance correction (AUC 0.77; ECE 0.006).

Models trained on T1 with BASE features showed poor forward generalization on T3b (AUC 0.32-0.38) despite high within-phase in-sample performance (Table S8 in Multimedia Appendix 1). This pattern is consistent with concept drift: the decline in abandonment rate from T1 to T3b, driven by skill consolidation and staffing adjustments, fundamentally altered the relationships between predictive features and outcomes. Adding operational metrics improved T1-trained models to AUC 0.715 (CatBoost) and 0.549 (random forest) on T3b, still well below their T2-trained counterparts, and with greater variability across the 4 test phases (SD 0.099 for CatBoost T1/with_agents vs 0.051 for the corresponding T2-trained model; Table S8 in Multimedia Appendix 1).

Across all temporal test sets (T1, T2, T3a, and T3b), T2-trained CatBoost models with operational features (unweighted and SMOTE variants) achieved AUC values of 0.73-0.85 and maintained consistent calibration (Figure S2 in Multimedia Appendix 1). Top-performing models achieved PR-AUC values of 0.068-0.071 on T3b, representing 2.47-2.58× lift over the T3b prevalence baseline of 0.0276 (Table S6 in Multimedia Appendix 1).

In contrast, Erlang-A (PR-AUC=0.031; 1.1× lift) and temporal heuristic (0.034; 1.2× lift) baselines achieved minimal discrimination above chance. The model’s value lies in ranking calls by risk to enable targeted interventions rather than requiring high absolute precision at any single threshold (Figure S4 in Multimedia Appendix 1).

### Model Interpretability and Feature Importance

**Figure 5:**
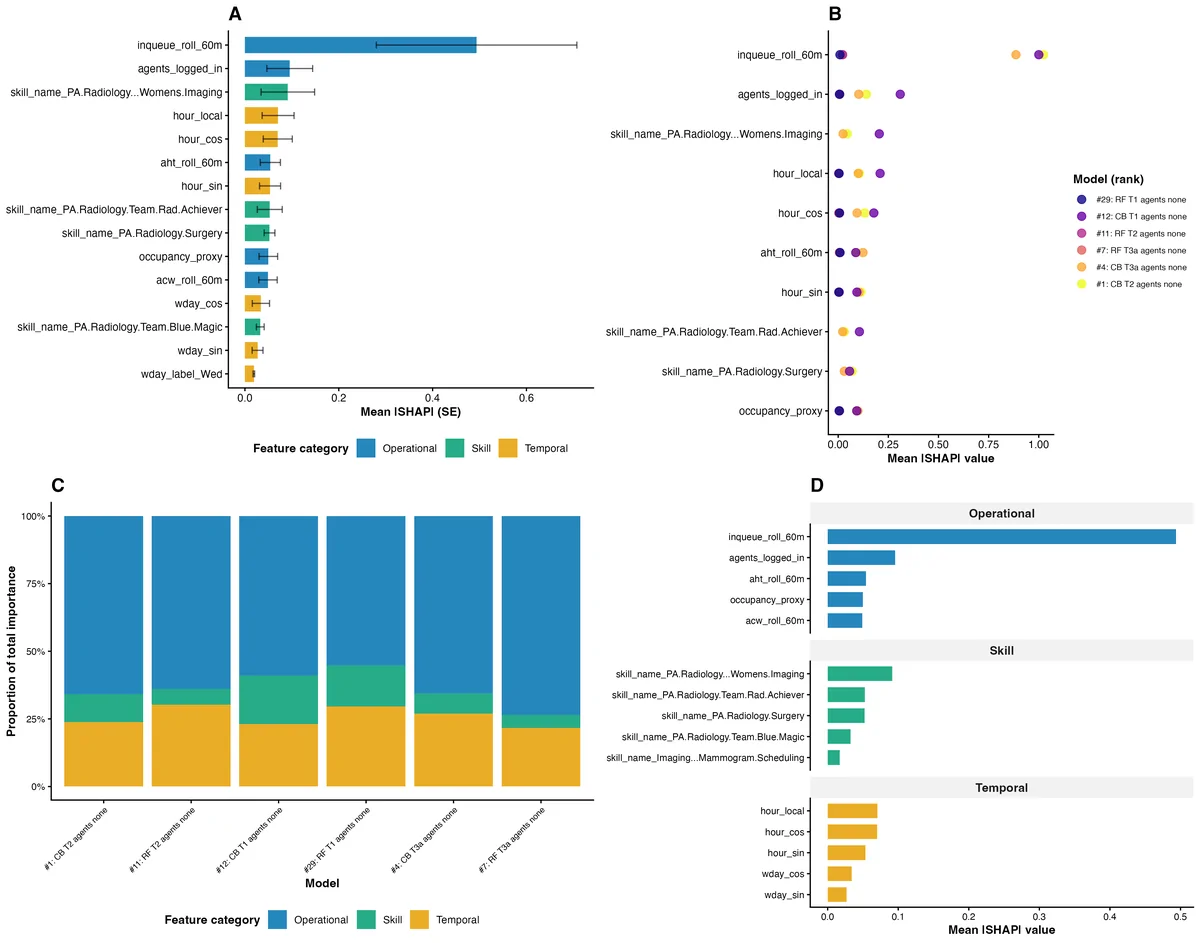
Shapley additive explanations (SHAP) feature importance for top-performing models: SHAP-based interpretability analysis across the 6 primary configurations (random forest and CatBoost trained on T1, T2, and T3a with operational features, no imbalance correction), the same set summarized in Table 3. (A) Ranking of the top 15 features by mean absolute SHAP value, revealing in-queue time as the dominant predictor (mean |SHAP| 0.494, SD 0.524, approximately 5 times the second-ranked feature, the number of agents logged in), followed by skill identifiers, hour-of-day encodings, and the remaining rolling operational metrics. (B) Comparison of feature importance across the 6 models individually, demonstrating that in-queue time’s dominance is consistent across training phases and algorithms. (C) Breaks down of each model’s total attributed importance by feature category (operational metrics, temporal patterns, skill identifiers): operational features account for approximately 64% (range 55%-74%) of total importance on average. (D) Ranking of the leading features within each category, showing that in-queue time and agent staffing lead the operational category by a wide margin.

SHAP analysis identified in-queue time as the dominant predictor, with a mean absolute SHAP value of 0.494 compared with 0.096 for the second-ranked feature (number of agents logged in; Figure 5; Table 3). Agent staffing, skill identifiers, and hour-of-day features occupied the next ranks, with the remaining rolling operational metrics (average handle time, occupancy rate, and after-call work time) closely behind; in-queue time’s dominance was consistent across training phases and algorithms (Figure S5 in Multimedia Appendix 1). The missingness-indicator variables introduced by the corrected feature specification carried negligible importance (mean |SHAP| ≤0.004), confirming that predictive signal does not derive from missingness patterns.

Temporal features (hour and day encoded via sine/cosine) showed lower SHAP magnitudes than in-queue time and agent staffing in all configurations, and skill identifiers appeared inconsistently and with lower importance (Figure 5; Figure S5 in Multimedia Appendix 1). Per-observation SHAP waterfall analysis illustrates feature directionality for representative T3b calls scored by the CatBoost T3a/with_agents configuration (Figure S10 in Multimedia Appendix 1). For a high-risk call (P[abandon]=0.727; actual: abandoned), the dominant drivers were routing to the specialized PA Interventional Radiology queue (SHAP=+1.3), a late-evening arrival (hour 23, SHAP=+0.8), zero observed occupancy (occupancy_proxy=0, SHAP=+0.7), and no agents observed answering within the prior hour (agents_logged_in=0, SHAP=+0.3). A borderline answered call (P[abandon]=0.010) showed offsetting contributions, with elevated recent queue time increasing risk (inqueue_roll_60m=50.5 seconds, SHAP=+0.6) offset by an off-peak arrival hour (SHAP=–0.3) and adequate staffing (agents_logged_in=13, SHAP=-0.2). Conversely, a low-risk call (P[abandon] <.001; actual: answered) was driven by short recent queue time (inqueue_roll_60m=2.4 seconds, SHAP=–1.4), zero after-call work in the window (SHAP=–0.7), and low occupancy (SHAP=–0.6). These examples confirm that the model’s decision logic aligns with operational intuition.

**Table 3:** Feature importance rankings (mean absolute Shapley additive explanations [SHAP] values): top 15 features ranked by mean absolute SHAP value across top-performing models. Columns include rank, feature name, feature category (operational, temporal, or skill), mean |SHAP| value, SD, and coefficient of variation (CV). In-queue time dominates as the most important predictor (mean |SHAP| 0.494, SD 0.524), followed by the number of agents logged in, skill identifiers, and hour-of-day features, with the remaining rolling operational metrics closely behind. In-queue time and agent staffing—both operational metrics—occupy the top 2 ranks, underscoring the primacy of queue-state information for abandonment prediction.

Rank	Feature	Category	Mean |SHAP| (SD)	CV	T1 |SHAP|	T2 |SHAP|	T3a |SHAP|	Models, n
1	inqueue_roll_60m	Operational	0.494 (0.524)	1.06	0.505	0.522	0.454	6
2	agents_logged_in	Operational	0.096 (0.119)	1.25	0.158	0.073	0.055	6
3	skill_name_PA.Radiology...Womens.Imaging	Skill	0.091 (0.099)	1.08	0.205	0.045	0.024	3
4	hour_local	Temporal	0.071 (0.083)	1.18	0.107	0.054	0.051	6
5	hour_cos	Temporal	0.070 (0.076)	1.08	0.092	0.069	0.048	6
6	aht_roll_60m	Operational	0.054 (0.053)	0.97	0.048	0.049	0.065	6
7	hour_sin	Temporal	0.054 (0.055)	1.02	0.048	0.059	0.053	6
8	skill_name_PA.Radiology.Team.Rad.Achiever	Skill	0.053 (0.046)	0.87	0.106	0.030	0.023	3
9	skill_name_PA.Radiology.Surgery	Skill	0.052 (0.020)	0.38	0.057	0.070	0.031	3
10	occupancy_proxy	Operational	0.050 (0.048)	0.97	0.049	0.048	0.053	6
11	acw_roll_60m	Operational	0.049 (0.048)	0.98	0.050	0.046	0.051	6
12	wday_cos	Temporal	0.034 (0.045)	1.32	0.019	0.023	0.060	6
13	skill_name_PA.Radiology.Team.Blue.Magic	Skill	0.033 (0.014)	0.44	0.037	0.017	0.045	3
14	wday_sin	Temporal	0.027 (0.028)	1.05	0.023	0.032	0.025	6
15	wday_label_Wed	Temporal	0.019 (0.003)	0.14	0.019	0.016	0.022	3

### Hyperparameter Tuning Impact

Hyperparameter tuning yielded marginal changes relative to default configurations for with_agents models (–0.008 to +0.015 ROC-AUC; maximum gain: CatBoost T3a/with_agents, 0.760 to 0.774), with 1 larger decline for a base configuration already performing below chance (CatBoost T1/base, –0.059). CatBoost selected depth=8 (matching the default) for the 3 base configurations but depth=10 for all 3 with_agents configurations. The performance advantage of CatBoost over random forest persisted after tuning (0.774 vs 0.754 for best with_agents models), confirming that the difference reflects algorithmic architecture rather than default parameter suitability (Figures S13a-S13c in Multimedia Appendix 1).

### Robustness Checks

Blocked temporal CV consistently produced lower performance estimates (mean delta: –0.079 ROC-AUC) than standard 5-fold CV, confirming some temporal autocorrelation within phases. Model ranking was preserved—CatBoost with agent features remained the top performer under both schemes—although with_agents configurations trained on the shorter posttransition phases scored 0.656-0.663 under blocked CV, indicating that standard CV materially overstates within-phase performance for these models. Our primary evaluation on the T3b holdout set, which inherently respects temporal ordering, provides the definitive performance assessment (Figure S11 and Table S4 in Multimedia Appendix 1).

Cost sensitivity analysis showed that at conventional false-negative to false-positive cost ratios (up to 11:1) the cost-minimizing policy was to flag no calls, because the model’s precision at practical operating points (approximately 5%-8%) does not offset the cost of FPs; flagging became cost-effective only at ratios of 12:1 and above (eg, a threshold of 0.08 achieving 22.1% sensitivity at 12:1, rising to 72.9% at 30:1). Cost-based deployment of this model is therefore appropriate only where a missed abandonment is weighted at least an order of magnitude more heavily than an unnecessary intervention (Figure S8 in Multimedia Appendix 1).

### Operational Patterns and Statistical Analysis

Skill-level analyses showed substantial variation across phases. The highest-volume skill (PA Radiology-Womens Imaging) accounted for 366,542 calls (35% of volume) and exhibited an abandonment decline from 13.9% in T1 to 0.5% in T3b (Table 4; Figure S6 in Multimedia Appendix 1). Consolidated high-volume skills showed transient increases during T2 before decreasing in T3b, while lower-volume specialized skills showed higher and more variable abandonment rates (>20% in T1). Across phases, the 3 highest-volume skills accounted for 73%-84% of total call volume.

Temporal patterns were consistent across operational phases (Tables S1a and S1b in Multimedia Appendix 1). Mondays had the highest abandonment rates (5274/48,411, 10.9% in T1, decreasing to 1999/58,049, 3.4% in T3b). Peak abandonment occurred from 11:00-16:00, coinciding with peak call volumes, while early morning and late afternoon periods showed lower abandonment rates. Operational metrics showed positive correlations with abandonment for in-queue time (*r*=0.18-0.31) and occupancy (*r*=0.03-0.07), and small negative correlations for handle time and after-call work time (*r*=–0.03 to –0.18).

**Table 4:** Skill-level performance matrix: summary of call volume and abandonment rates for major skills across all 4 operational phases. For each skill, the table shows total calls and abandonment percentage in T1, T2, T3a, and T3b. The matrix reveals heterogeneous adaptation patterns, with the highest-volume skill (PA Radiology-Womens Imaging) showing dramatic improvement from 13.9% abandonment in T1 to 0.5% in T3b, while consolidated skills showed varied trajectories including transient increases during the transitional period.

Skill name	Total	T1 (abandon), n (%)	T2 (abandon), n (%)	T3a (abandon), n (%)	T3b (abandon), n (%)
PA Radiology-Womens Imaging	366,542	90,167 (13.9)	93,066 (5.2)	81,169 (2.6)	102,140 (0.5)
PA Radiology Team Rad Achiever	256,641	48,490 (1.6)	68,081 (6.3)	57,656 (3.3)	82,414 (4.9)
PA Radiology Team Blue Magic	189,344	32,695 (1.6)	50,764 (6.7)	43,206 (2.5)	62,679 (4.3)
PA Radiology Surgery	89,275	14,614 (2.8)	24,988 (1.9)	20,554 (1.1)	29,119 (1.3)
PA Interventional Radiology	53,182	11,819 (2.4)	13,031 (2.1)	11,461 (2.3)	16,871 (3)
Imaging-Mammogram Scheduling	35,166	23,867 (23)	11,297 (6.6)	1 (0)	1 (100)
Imaging-CT Scheduling	12,570	7375 (1.1)	5188 (4)	0 (0)	7 (85.7)
PA Imaging Default	12,197	0 (0)	3817 (6.3)	3307 (3.4)	5073 (2.1)
Imaging-MRI Scheduling	7355	3718 (1.7)	3633 (4.4)	0 (0)	4 (50)
Imaging-Tech	6822	3131 (14.8)	3691 (9.8)	0 (0)	0 (0)
PA Imaging-Echo Campaign	6167	1771 (3.4)	1661 (1.1)	1242 (0.6)	1493 (2.3)
PA Radiology-IR RN	1463	460 (13.3)	355 (12.4)	389 (15.2)	259 (10.4)
PA Radiology-PET Scan	639	615 (3.9)	24 (12.5)	0 (0)	0 (0)

### Threshold Optimization

Seven threshold optimization policies were evaluated, each mapping to a specific deployment scenario [16] (Figure S7 in Multimedia Appendix 1). These included *F*_1_-maximizing (balanced precision-recall), Youden J (sensitivity-specificity balance), cost-minimizing under the assumed 5:1 FN:FP ratio, precision-targeted at 95%, recall-targeted at 90%, volume-based (top 10% risk decile), and maximum false positive rate constrained at 5%. The *F*_1_-maximizing threshold was approximately 0.06, identifying about 14% (43,063/300,060) of calls as positive predictions. Youden-index optimization produced a threshold near 0.02. Under the assumed 5:1 false-negative-weighted cost model, cost minimization favored flagging no calls, because the model’s precision at this prevalence does not offset false-positive costs at that ratio (Figure S8 in Multimedia Appendix 1).

Target-recall optimization for 90% abandonment sensitivity required a threshold near 0.012, flagging approximately 52% (157,052/300,060) of calls (sensitivity 90.0%, 7463 of 8293 positives correctly identified; specificity 48.8%; precision 0.047). At threshold 0.03, the same model achieves sensitivity of 72.9% (6045/8293 positives) at a lower flagging volume of 32.2% (96,626/300,060), illustrating the recall-volume trade-off inherent to rare-event detection at low prevalence. Operating thresholds also transfer prospectively: under a Youden-index policy, the threshold derived on T3a (0.03) achieves 72.9% sensitivity on T3b vs 82.1% for the T3b-optimal threshold (0.02), and under the 90%-sensitivity-target policy the T3a-derived threshold coincides with the T3b-optimal threshold.

## Discussion

### Principal Findings

This study set out with a fundamental question: could call abandonment in health care call centers be predicted using machine learning in conjunction with nonpersonal operational data? This question emerged from the pressing need to balance operational efficiency with increasingly stringent patient privacy requirements in health care settings [6,7]. Using an extensive longitudinal dataset spanning 17 months and comprising over 1.2 million calls from Emory Healthcare’s call center, we explored the feasibility of abandonment prediction across distinct operational phases marked by significant organizational transitions. By leveraging random forest and CatBoost algorithms combined with interpretable machine learning approaches, we aimed to develop a privacy-compliant framework capable of adapting to evolving call center conditions while maintaining robust predictive power [10].

Our findings demonstrate that call abandonment can be accurately predicted using exclusively nonpersonal operational metrics, with real-time queue dynamics emerging as the most influential determinants. These metrics, particularly in-queue time and agent staffing, consistently carried substantially more predictive signal than temporal patterns or skill assignments. This highlights that abandonment behavior reflects immediate system conditions rather than fixed characteristics of callers or services. Importantly, models trained on data reflecting the current operational structure retained strong discrimination and calibration, whereas models trained on preintervention data showed substantial degradation when applied prospectively. This reinforces that organizational shifts, such as skill consolidation or workflow restructuring, fundamentally reshape relationships between features and outcomes, and therefore predictive models must be retrained following operational change.

### Comparison With Prior Work

These observations align closely with foundational queueing theory, which posits that customer abandonment is driven primarily by perceived waiting burden and system congestion rather than arrival patterns or service categories [1,8]. Our results extend this theory into the health care domain, providing large-scale empirical validation using machine learning rather than stylized analytical models. They also support prior evidence that predictive model performance in health care systems is sensitive to workflow changes and organizational redesign [1,9]. Notably, our work adds nuance to this literature by showing that models trained during transitional periods, typically avoided by practitioners, can generalize effectively, likely because transitional variability exposes algorithms to a wider distribution of operational conditions, consistent with robustness research in uncertain environments [2].

Skill-level analysis revealed that while aggregate abandonment rates improved following consolidation, this improvement was not uniform. High-volume core skills such as “PA Radiology-Womens Imaging” showed substantial reductions in abandonment, while consolidated queues including “PA Radiology Team Rad Achiever” and “Team Blue Magic” experienced transient worsening during the transitional period before partial stabilization in T3b. SHAP analysis corroborates this heterogeneity, with certain specialized skill queues consistently appearing as top abandonment risk drivers. These findings suggest that consolidation strategies may create unintended bottlenecks in specialized service lines, and flexible surge capacity may be needed to accommodate adjustment-related increases.

### Operational Implications

A key conceptual contribution of this study is demonstrating that strong predictive performance does not require patient demographics or identifiable behavioral histories. Traditional customer service analytics frequently rely on such data, but these features introduce substantial privacy and compliance barriers in health care contexts [6,7]. By showing that nonpersonal operational data alone is sufficient for robust prediction, we provide health care organizations with a viable and compliant pathway for implementing predictive analytics. The interpretability of the resulting models reinforces their practical use: unlike demographic predictors, operational metrics translate directly into actionable interventions that call center supervisors can modify, such as staffing adjustments, routing logic, or queue management policies. In practice, these capabilities support several deployment modes: real-time staffing alerts triggered when aggregate risk scores exceed queue-level thresholds, dynamic routing of high-risk calls toward less congested skill queues, and proactive callback offers via interactive voice response when predicted abandonment probability exceeds a configurable threshold. At a strategic level, historical patterns of risk by hour, day, and skill queue can inform staffing schedules and capacity planning. Each pathway carries different tolerance for FPs, which the threshold optimization policies described above are designed to accommodate.

The consistency of certain temporal patterns, such as higher abandonment on Mondays and peak loads during late morning and midday, further suggests that health care–seeking behavior follows stable rhythms independent of workflow changes. These patterns align with previous observations in health care operations research [3,5] and offer predictable levers for resource planning. Skill-level variation, however, points to the need for differentiated management strategies. High-volume core skills demonstrated stability and improvement, whereas lower-volume specialized skills showed greater volatility and higher baseline abandonment. This suggests that uniform staffing strategies may be suboptimal, and that flexible or surge capacity may be more effective for specialized services encountering sporadic but impactful variability. Importantly, even in the stabilized holdout environment, where low median queue times and reduced abandonment suggest a well-optimized system, the right-skewed distribution of queue conditions means that abandonment events cluster during peak hours and at the start of the work week, when transient capacity constraints emerge. The model’s practical value in such settings lies not in predicting routine calls, but in detecting these episodic windows of elevated risk through real-time operational signals rather than static temporal heuristics.

### Limitations

First, this study used data from a single health care system; although the underlying principles, such as the primacy of queue-based metrics and the need for retraining after workflow changes, are likely generalizable, specific performance metrics may differ in other contexts with distinct operational structures or patient populations [7]. Future multicenter studies would strengthen external validity.

Second, absolute precision remains modest at most operating thresholds, inherent to predicting a rare event at low prevalence. While lift over the prevalence baseline demonstrates meaningful discrimination, deployment must account for FP burden. The model is best suited for risk ranking and aggregate alert systems rather than individual-call binary classification.

Third, the consolidation of skill assignments during the study period introduces some confounding, as performance changes cannot be fully disentangled from structural reorganization. Relatedly, several legacy scheduling queues that were not part of the twelve-skill consolidation, “Imaging-Mammogram Scheduling” (23,867 calls in T1, declining to 1 call in T3a and 1 in T3b), “Imaging-CT Scheduling” (7375 calls in T1, 0 in T3a, and 7 in T3b), and “Imaging-MRI Scheduling” (3718 calls in T1, 0 in T3a, and 4 in T3b), dropped to near-zero call volume in the later phases as operations shifted away from them. This constitutes a form of feature drift: these queues contribute to model interpretability rankings despite carrying almost no signal in T3a/T3b, and interpretability findings attributable specifically to these queues should be interpreted in light of this volume collapse rather than as evidence of a stable, generalizable relationship.

The study period of 17 months, while spanning significant operational changes, may not encompass longer-term secular trends. Blocked temporal CV confirmed some temporal autocorrelation in within-phase estimates, though model ranking was preserved and the prospective holdout evaluation inherently respects temporal ordering.

Fourth, the absence of EWT announcements in our setting simplifies interpretation of in-queue time as a pure system-state indicator but limits generalizability. In settings where EWT announcements are provided, this information may represent a primary causal mechanism for abandonment and should be included as a feature in future multisite studies.

Fifth, calls are treated as independent observations. While individual callers may contribute multiple calls, the false abandonment analysis revealed that only a small fraction of abandoned calls involved callers who reconnected, suggesting limited within-caller dependence. Future work could explore mixed-effects models or clustered SEs to formally account for caller-level clustering.

### Future Directions

Future work should focus on multicenter validation to evaluate generalizability across diverse health care systems with varying operational structures [4]. Prospective real-time deployment studies will be essential to characterize the full intervention loop: prediction generation, operational responses, and impact on abandonment rates [9]. Integrating predictive models with workforce management platforms could enable automated staffing or routing adjustments in response to predicted congestion. Methodological extensions, such as reinforcement learning for optimal intervention policies, causal inference for identifying modifiable drivers, or privacy-preserving modeling architectures, could further strengthen applicability while maintaining compliance. Beyond abandonment prediction, applying similar frameworks to outcomes such as first-call resolution, appointment completion, or patient satisfaction could support a unified predictive ecosystem for health care access optimization.

For production deployment, we recommend (1) quarterly retraining on the most recent operational window to match current conditions, (2) continuous monitoring of calibration metrics on live predictions, (3) an alert trigger when rolling discrimination drops below an acceptable threshold to flag retraining needs, and (4) tracking feature distribution shifts (eg, changes in mean agent availability or queue congestion metrics) as early warning indicators of concept drift before performance degrades [17,23].

Decision-oriented evaluation frameworks, as advocated by Itzhak et al [16] for rare-event prediction under temporal dynamics, highlight the importance of matching evaluation metrics to deployment objectives. Our threshold optimization policies directly instantiate this principle, providing deployment-ready operating points rather than relying solely on aggregate discrimination metrics.

### Conclusions

Call abandonment in a health care call center can be predicted from nonpersonal operational data alone, with CatBoost trained on a postintervention phase achieving ROC-AUC 0.767 (95% CI 0.762-0.771) and PR-AUC 0.068 (2.47× lift over the T3b prevalence baseline 0.0276) on a 300,060-call temporal holdout. Real-time queue dynamics, particularly in-queue time and active agent count, provide the dominant predictive signal, and models trained on data reflecting current operational conditions generalize better to subsequent periods than models trained on preintervention data.

## Appendix

Multimedia Appendix 1 Additional material.

## Abbreviations

AUC: area under the curve
CV: cross-validation
ECE: expected calibration error
EWT: estimated wait time
FN: false negative
FP: false positive
HIPAA: Health Insurance Portability and Accountability Act
IRB: institutional review board
PR-AUC: precision-recall area under the curve
ROC-AUC: area under the receiver operating characteristic curve
SHAP: Shapley additive explanations
SMOTE: synthetic minority oversampling technique

## Data Availability

Data supporting the results reported in this manuscript are available from the corresponding author upon reasonable request. The call center data contain deidentified operational records provided for this analysis and cannot be publicly shared because they remain institutional operational data. The analysis code (15 R scripts covering data preparation, model training, temporal validation, threshold optimization, interpretability analysis, and figure generation) is available from the corresponding author upon reasonable request.
