# Supplementary material for: Predicting Call Abandonment in a Health Care Call Center Using Nonpersonal Operational Data: Machine Learning Study
Source: JMIR Med Inform. 2026 Aug 31;14:e88441. doi: 10.2196/88441 (PMC13575546; doi:10.2196/88441)
Supplement: Supplementary file 1 [file medinform_v14i1e88441_app1.docx]

**Supplementary Materials**

## Supplementary Figure S1

Supplementary Figure S1: Data Characteristics and Operational Context

Call volume distribution and operational characteristics across temporal and categorical dimensions. Panel A displays total call volume by operational phase, showing the dataset composition (T1: 238,722 calls, T2: 279,596 calls, T3a: 218,985 calls, T3b: 300,060 calls). Panel B presents call volume stratified by weekday and phase, revealing consistent weekly patterns with Monday showing highest volume across all periods. Panel C shows hourly call volume distribution, identifying peak demand during midday hours (11:00 AM to 4:00 PM) with characteristic bimodal patterns. Panel D illustrates the top 10 skills by call volume as percentage of total calls per phase, demonstrating the concentration of volume in high-volume radiology services and the effects of skill consolidation across operational periods.

## Supplementary Figure S2

Supplementary Figure S2: Model Calibration Analysis

Detailed calibration assessment evaluating how well predicted probabilities match observed abandonment rates for top-performing models. Panel A shows calibration curves for the top 6 models, plotting predicted probability against observed frequency across probability bins, with the diagonal line representing perfect calibration. Panel B presents calibration by decile with 95% confidence intervals, showing model reliability across the full probability range. Panel C displays reliability diagrams combining histogram of prediction distribution with calibration assessment, illustrating that most predictions fall in low-probability ranges consistent with the 2.8% baseline abandonment rate. Panel D compares calibration metrics (Expected Calibration Error [ECE] and Maximum Calibration Error) across models, demonstrating that the recommended model achieves ECE = 0.006 (Brier 0.027), and unweighted and SMOTE variants of the top CatBoost configurations achieve ECE ≤ 0.011, while class-weighted variants show substantially degraded calibration.

## Supplementary Figure S3

Supplementary Figure S3: Temporal Drift Analysis

Analysis of temporal drift patterns and feature importance stability across operational phases. Panel A tracks model performance (Area Under the Curve [AUC]) across all test phases for models grouped by training phase, illustrating performance degradation patterns when models are tested on data from different operational regimes. Lines show individual model trajectories, with T1-trained models showing sharp performance drops on post-intervention phases. Panel B examines feature importance stability across training phases (T1, T2, T3a), showing that operational metrics maintain consistent importance rankings despite organizational changes, while skill-specific features show high variability reflecting skill consolidation efforts. Panel C displays distribution shifts in key operational features (in-queue time, occupancy, Average Handle Time [AHT]) over phases using violin plots, quantifying the magnitude of operational changes between pre- and post-intervention periods. Panel D presents a phase-to-phase performance transition matrix showing AUC values when models trained on one phase are tested on another, highlighting the asymmetric degradation where historical models fail on recent data but recent models maintain reasonable performance on historical data.

## Supplementary Figure S4

Supplementary Figure S4: Precision-Recall Analysis

Comprehensive Precision-Recall (PR) analysis for the imbalanced classification problem (2.8% abandonment rate in holdout phase). Panel A displays PR curves for the top 10 models evaluated on the T3b holdout set, with PR-AUC values ranging from 0.061 to 0.071. The horizontal dashed line indicates baseline prevalence (no-skill classifier). Panel B compares PR-AUC across algorithms and feature sets, demonstrating consistent improvements from operational features regardless of algorithm choice. Panel C illustrates the precision-recall tradeoff at different probability thresholds (0.01, 0.03, 0.05, 0.10, 0.20), showing that low thresholds achieve high recall but low precision, while high thresholds achieve high precision but low recall. Panel D shows F1 score optimization, identifying optimal thresholds that balance precision and recall for different operational priorities, with a peak F1 score of approximately 0.13 achieved at thresholds around 0.05-0.07.

## Supplementary Figure S5

Supplementary Figure S5: Detailed SHAP Feature Importance Analysis

Extended SHapley Additive exPlanations (SHAP) analysis examining feature importance patterns across model configurations and temporal phases. Panel A compares mean absolute SHAP values between algorithms (Random Forest vs. CatBoost) for matched model configurations on the T3b holdout, showing strong concordance for models trained on post-transition phases (Pearson correlation 0.95 for T2- and T3a-trained pairs) and weaker concordance for T1-trained pairs (0.61), consistent with operational drift from the earliest phase; in-queue time is the dominant feature under both algorithms. Panel B tracks feature importance across training phases (T1, T2, T3a) by category, showing that in-queue time remains dominant in every phase while most features peak in importance for T1-trained models. Panel C displays top features by category with variance bars, quantifying both mean importance and stability across models; the missingness-indicator variables introduced by the corrected feature specification rank at the bottom of the operational category with negligible importance. Panel D ranks features by mean absolute SHAP value with each point colored by its coefficient of variation across models (approximately 0.4-1.3 for the top-ranked features), showing that the most important features are also among the most stable.

## Supplementary Figure S6

Supplementary Figure S6: Skill-Level Performance Heterogeneity

Variation in abandonment rates and operational characteristics across service skills. Panel A presents a heatmap of abandonment rates for the five skills with the highest and the five with the lowest mean abandonment rates across the four operational phases, revealing heterogeneous responses to organizational interventions. Each cell is annotated with the abandonment rate and the number of calls (n); grey cells marked “No calls” are phases in which the queue received no calls, and rates with very small denominators (for example, 100.0% with n=1) arise from the retired legacy scheduling queues discussed in the Limitations. Color intensity indicates abandonment rate, with darker colors representing higher abandonment. Panel B displays abandonment rate trajectories for skills showing the largest changes between T1 and T3b, illustrating different adaptation patterns: dramatic improvements (e.g., PA Radiology - Womens Imaging declining from 13.9% to 0.5%), transient increases during transition (consolidated skills showing temporary elevation in T2), and persistent high abandonment in low-volume specialized services. Panel C compares skill-specific operational metrics (mean in-queue time, AHT, occupancy rate) across phases, showing the operational mechanisms underlying abandonment differences. Panel D shows the distribution of abandonment rates across all active skills by phase, demonstrating overall improvement and reduced variability over time.

## Supplementary Figure S7

Supplementary Figure S7: Threshold Analysis and Operating Characteristics

Threshold analysis for the recommended model (CatBoost T2/with_agents/none) on the T3b holdout. Panel A displays sensitivity (recall), specificity, Positive Predictive Value (PPV), and Negative Predictive Value (NPV) as functions of the classification threshold, with reference lines at thresholds of 1%, 3%, 5%, and 10%; lower thresholds increase sensitivity at the cost of PPV. Achieving 90% sensitivity requires a threshold near 0.012 and flags 52.3% of calls, while a threshold of 0.03 yields 72.9% sensitivity at 32.2% flagged. Panel B presents confusion matrices at the four reference thresholds, annotated with per-class rates. Panel C displays sensitivity, specificity, and F1 score as functions of the threshold; F1 peaks at approximately 0.13 near a threshold of 0.06. Panel D summarizes specificity, sensitivity, PPV, NPV, and F1 at the 5% and 10% thresholds, illustrating the operating characteristics available for deployment decisions at low prevalence (2.8%).

## Supplementary Figure S8

Supplementary Figure S8: Cost Sensitivity Analysis

Effect of the assumed false-negative to false-positive (FN:FP) cost ratio on the cost-minimizing decision threshold and its operating characteristics, for the recommended model (CatBoost T2/with_agents/none) on the T3b holdout. At cost ratios up to 11:1, the cost-minimizing policy is to flag no calls (threshold above the maximum predicted probability; sensitivity 0%): with precision at practical operating points between 5% and 8%, the expected cost of acting on false positives exceeds the expected savings from prevented abandonments. Flagging becomes cost-effective at FN:FP ratios of 12:1 and above (threshold 0.08, sensitivity 22.1%, precision 8.2%), with sensitivity rising to 50.8% at 15:1 and 72.9% at 30:1. Cost-based deployment of this model is therefore appropriate only where a missed abandonment is judged at least an order of magnitude more costly than an unnecessary intervention; otherwise, sensitivity-target policies (Supplementary Figure S7) are the appropriate operating mode.

## Supplementary Figure S9

Supplementary Figure S9: Machine Learning Versus Baseline Model Performance

Machine learning versus baseline model performance on the T3b holdout. Panel A (ROC-AUC): CatBoost (0.767) and Random Forest (0.749) outperform Logistic Regression (0.736), which substantially outperforms Erlang-A (0.561), the temporal heuristic (0.562), and the prevalence-only baseline (0.500). Panel B (PR-AUC): ML models achieve up to 2.58× lift over the T3b prevalence baseline (0.0276; 2.47× for the recommended configuration), while the queueing and heuristic baselines achieve approximately 1.1-1.2× lift.

## Supplementary Figure S10

Supplementary Figure S10: Individual SHAP Waterfall Explanations for Representative Calls. Per-observation SHAP waterfalls for three representative T3b calls scored by the CatBoost T3a/with_agents/none configuration, with per-feature contributions to predicted log-odds beginning from the model's base log-odds of −4.252; orange bars increase abandonment risk and blue bars decrease it. Panel A: high-risk call with P(abandon) = 0.727 (actually abandoned), dominated by specialized skill routing to PA Interventional Radiology (SHAP = +1.3), a late-evening arrival (hour 23, SHAP = +0.8), zero observed occupancy (SHAP = +0.7), and no agents observed answering within the prior hour (SHAP = +0.3). Panel B: borderline call with P(abandon) = 0.010 (actually answered), illustrating offsetting contributions — elevated rolling in-queue time (50.5 s, SHAP = +0.6) offset by an off-peak arrival hour (SHAP = −0.3) and adequate staffing (13 agents logged in, SHAP = −0.2). Panel C: low-risk call with P(abandon) < 0.001 (actually answered), driven by short rolling in-queue time (2.4 s, SHAP = −1.4), zero after-call work in the window (SHAP = −0.7), and low occupancy (SHAP = −0.6).

## Supplementary Figure S11

Supplementary Figure S11: Blocked Temporal Cross-Validation

Expanding-window blocked temporal CV versus standard 5-fold CV. Panel A (ROC-AUC): Blocked CV consistently yields lower estimates (mean delta: −0.079; all 12 deltas negative) across all 12 algorithm-phase-feature configurations, but preserves model ranking. Panel B (Brier score): Calibration differences between CV methods are smaller, indicating temporal autocorrelation primarily affects discrimination estimates.

## Supplementary Figure S12

Supplementary Figure S12: False Abandonment Sensitivity Analysis

Sensitivity analysis for false abandonment exclusion. Panel A: ROC-AUC on T3b as callers who reconnected within 1h, 4h, 12h, and 24h windows are progressively excluded, with 95% confidence band. Performance changes by only +0.003. Panel B: Percentage of T3b calls excluded at each window (all <0.05%). Panel C: False abandonment rate by phase and reconnection window (1.2%-1.7% of abandoned calls across all phases).

## Supplementary Figure S13a

Supplementary Figure S13a: Hyperparameter Tuning Impact

Default versus grid-searched hyperparameters on the T3b holdout set. Panel A: Side-by-side ROC-AUC for each algorithm-phase-feature combination under default and tuned settings. Panel B: Delta ROC-AUC (tuned minus default); positive values indicate tuning improved performance. Deltas are ≤0.015 in magnitude except CatBoost T1/base (−0.059); tuning does not change the recommended configuration. Tuned T1/base models remain below chance on the T3b holdout (CatBoost 0.316, RF 0.324), reflecting temporal drift rather than tuning failure.

## Supplementary Figure S13b

Supplementary Figure S13b: Random Forest Hyperparameter Grid Search

Heatmaps show mean 3-fold inner CV ROC-AUC across mtry (number of candidate split features) and min_n (minimum node size) for each training phase (T1, T2, T3a) and feature set (base, with_agents). Darker cells indicate higher AUC. Performance is stable across the grid, with with_agents models outperforming base by 0.03-0.13 AUC in best inner-CV performance (T1: +0.03; T2: +0.13; T3a: +0.09).

## Supplementary Figure S13c

Supplementary Figure S13c: CatBoost Hyperparameter Grid Search

Heatmaps show mean 3-fold inner CV ROC-AUC across tree depth (4, 6, 8, 10) and learning rate (0.01, 0.05, 0.1) for each training phase and feature set. Depth=8 with learning_rate=0.01 remains optimal for the three base configurations; all three with_agents configurations instead select depth=10 (learning_rate 0.01 for T1/T3a, 0.05 for T2), indicating that deeper trees better exploit the corrected operational features.

# Supplementary Tables

## Supplementary Table S1a

Supplementary Table S1a: Hourly Abandonment Patterns Across Operational Phases

Call abandonment rates and call volumes by hour of day across all four operational phases (T1, T2, T3a, T3b). For each hour (0-23), the table shows the number of abandoned calls, total calls, and abandonment percentage. Peak abandonment occurs during midday hours (11:00 AM to 4:00 PM) across all phases, with rates ranging from 7-13% in T1 declining to 2-6% in T3b. Early morning (8:00-9:00 AM) and evening hours (5:00-7:00 PM) show substantially lower abandonment despite moderate call volumes, indicating adequate capacity during these periods. These temporal patterns remain consistent across all operational phases despite overall abandonment declining 68% from T1 to T3b. Rows for hours 01:00 through 06:00 are zero-call hours during which the call center is closed; they are shown for completeness. Each cell reports abandoned calls/total calls (abandonment rate); cells shown as 0/0 (—) are periods in which the phase recorded no calls, distinguishing zero-call periods from zero-abandonment periods.

| Hour | T1 | T2 | T3a | T3b |
| --- | --- | --- | --- | --- |
| 00:00 | 0/0 (—) | 0/0 (—) | 1/53 (1.9%) | 5/219 (2.3%) |
| 01:00 | 0/0 (—) | 0/0 (—) | 0/0 (—) | 0/0 (—) |
| 02:00 | 0/0 (—) | 0/0 (—) | 0/0 (—) | 0/0 (—) |
| 03:00 | 0/0 (—) | 0/0 (—) | 0/0 (—) | 0/0 (—) |
| 04:00 | 0/0 (—) | 0/0 (—) | 0/0 (—) | 0/0 (—) |
| 05:00 | 0/0 (—) | 0/0 (—) | 0/0 (—) | 0/0 (—) |
| 06:00 | 0/0 (—) | 0/0 (—) | 0/0 (—) | 0/0 (—) |
| 07:00 | 0/0 (—) | 0/11 (0.0%) | 1/12 (8.3%) | 0/0 (—) |
| 08:00 | 331/19163 (1.7%) | 169/14204 (1.2%) | 73/10233 (0.7%) | 0/7 (0.0%) |
| 09:00 | 1396/28060 (5.0%) | 566/19762 (2.9%) | 251/14201 (1.8%) | 0/6 (0.0%) |
| 10:00 | 2411/30964 (7.8%) | 991/21217 (4.7%) | 369/15212 (2.4%) | 0/18 (0.0%) |
| 11:00 | 2926/30025 (9.7%) | 1408/20590 (6.8%) | 654/14350 (4.6%) | 3/54 (5.6%) |
| 12:00 | 3528/26254 (13.4%) | 1715/29122 (5.9%) | 385/17948 (2.1%) | 164/14696 (1.1%) |
| 13:00 | 3278/26536 (12.4%) | 1945/33350 (5.8%) | 558/24354 (2.3%) | 754/32606 (2.3%) |
| 14:00 | 3042/27394 (11.1%) | 2834/34930 (8.1%) | 799/26486 (3.0%) | 1392/39632 (3.5%) |
| 15:00 | 2425/26830 (9.0%) | 2469/33622 (7.3%) | 867/26009 (3.3%) | 1369/39587 (3.5%) |
| 16:00 | 1406/20721 (6.8%) | 1025/25591 (4.0%) | 519/21282 (2.4%) | 1274/35561 (3.6%) |
| 17:00 | 51/1468 (3.5%) | 512/12029 (4.3%) | 338/12508 (2.7%) | 976/31741 (3.1%) |
| 18:00 | 8/824 (1.0%) | 717/12602 (5.7%) | 498/11777 (4.2%) | 926/31388 (3.0%) |
| 19:00 | 7/482 (1.5%) | 501/12173 (4.1%) | 277/11586 (2.4%) | 735/31009 (2.4%) |
| 20:00 | 0/1 (0.0%) | 197/9094 (2.2%) | 107/9329 (1.1%) | 337/26697 (1.3%) |
| 21:00 | 0/0 (—) | 46/691 (6.7%) | 19/3197 (0.6%) | 187/13168 (1.4%) |
| 22:00 | 0/0 (—) | 24/452 (5.3%) | 7/321 (2.2%) | 128/2790 (4.6%) |
| 23:00 | 0/0 (—) | 3/156 (1.9%) | 2/127 (1.6%) | 43/881 (4.9%) |

## Supplementary Table S1b

Supplementary Table S1b: Daily Abandonment Patterns Across Operational Phases

Call abandonment rates and call volumes by day of week across all four operational phases (T1, T2, T3a, T3b). For each weekday (Monday through Sunday), the table shows the number of abandoned calls, total calls, and abandonment percentage. Monday consistently exhibits the highest abandonment rates across all phases (10.9% in T1 declining to 3.4% in T3b), with rates 20-40% higher than other weekdays even as absolute rates decline. This persistent Monday effect suggests fundamental demand-capacity misalignments at week starts that persisted despite overall operational improvements. Each cell reports abandoned calls/total calls (abandonment rate); cells shown as 0/0 (—) are periods in which the phase recorded no calls, distinguishing zero-call periods from zero-abandonment periods.

| Day of Week | T1 | T2 | T3a | T3b |
| --- | --- | --- | --- | --- |
| Mon | 5274/48411 (10.9%) | 3828/58441 (6.6%) | 1839/45686 (4.0%) | 1999/58049 (3.4%) |
| Tue | 4291/49864 (8.6%) | 2476/53912 (4.6%) | 887/45221 (2.0%) | 1292/61036 (2.1%) |
| Wed | 3639/47976 (7.6%) | 2957/55206 (5.4%) | 709/44653 (1.6%) | 1557/61283 (2.5%) |
| Thu | 3573/46724 (7.6%) | 2620/58090 (4.5%) | 888/42486 (2.1%) | 1703/60856 (2.8%) |
| Fri | 4032/45747 (8.8%) | 3240/53944 (6.0%) | 1387/40501 (3.4%) | 1678/56979 (2.9%) |
| Sat | 0/0 (—) | 0/1 (0.0%) | 10/357 (2.8%) | 61/1506 (4.1%) |
| Sun | 0/0 (—) | 1/2 (50.0%) | 5/81 (6.2%) | 3/351 (0.9%) |

## Supplementary Table S1c

Supplementary Table S1c: Monthly Abandonment Patterns Across Operational Phases

Call abandonment rates and call volumes by month across the study period (January 2023 through May 2024). For each month, the table shows the number of abandoned calls, total calls, and abandonment percentage. The table reveals seasonal patterns and the impact of organizational interventions, with abandonment rates declining substantially following the intervention period (May-August 2023). Monthly call volumes show characteristic patterns with peaks in certain months and lower volumes during traditional holiday periods. Each cell reports abandoned calls/total calls (abandonment rate); cells shown as 0/0 (—) are periods in which the phase recorded no calls, distinguishing zero-call periods from zero-abandonment periods.

| Month | T1 | T2 | T3a | T3b |
| --- | --- | --- | --- | --- |
| Jan | 3282/53883 (6.1%) | 0/0 (—) | 0/0 (—) | 857/58662 (1.5%) |
| Feb | 4410/55491 (7.9%) | 0/0 (—) | 0/0 (—) | 1177/58836 (2.0%) |
| Mar | 8801/72350 (12.2%) | 0/0 (—) | 0/0 (—) | 1939/59341 (3.3%) |
| Apr | 4316/56998 (7.6%) | 0/0 (—) | 0/0 (—) | 2253/61120 (3.7%) |
| May | 0/0 (—) | 4549/64583 (7.0%) | 0/0 (—) | 2067/62101 (3.3%) |
| Jun | 0/0 (—) | 3478/64602 (5.4%) | 0/0 (—) | 0/0 (—) |
| Jul | 0/0 (—) | 5033/82080 (6.1%) | 0/0 (—) | 0/0 (—) |
| Aug | 0/0 (—) | 2062/68331 (3.0%) | 0/0 (—) | 0/0 (—) |
| Sep | 0/0 (—) | 0/0 (—) | 1423/56616 (2.5%) | 0/0 (—) |
| Oct | 0/0 (—) | 0/0 (—) | 2002/61379 (3.3%) | 0/0 (—) |
| Nov | 0/0 (—) | 0/0 (—) | 1489/54331 (2.7%) | 0/0 (—) |
| Dec | 0/0 (—) | 0/0 (—) | 811/46659 (1.7%) | 0/0 (—) |

## Supplementary Table S2

Supplementary Table S2: False Abandonment Sensitivity Analysis

Impact of excluding false abandonments (callers who abandoned but reconnected within specified time windows) on model performance. The table shows ROC-AUC, PR-AUC, Brier score, and ECE for the model used for the interpretability and sensitivity analyses (CatBoost T3a/with_agents/none) on the T3b holdout set under different exclusion scenarios. All reconnections occur within the first hour; extending the window to 4, 12, or 24 hours identifies no additional false abandonments. Excluding these 143 calls (0.05% of T3b) changes ROC-AUC by only +0.003, confirming negligible impact on model performance.

Panel A. False abandonment prevalence by phase

| Phase | N abandoned | N false (1h) (identical at 4h, 12h, 24h) | % false |
| --- | --- | --- | --- |
| T1 | 20,809 | 276 | 1.33% |
| T2 | 15,122 | 202 | 1.34% |
| T3a | 5,725 | 70 | 1.22% |
| T3b | 8,293 | 143 | 1.72% |

Panel B. Impact on T3b model performance under different reconnection-window exclusions.

| scenario | window_hours | n_total | n_excluded | pct_excluded | roc_auc_ci | pr_auc_ci | brier_fmt | ece_fmt |
| --- | --- | --- | --- | --- | --- | --- | --- | --- |
| all_calls | 0 | 300060 | 0 | 0.00 | 0.760 [0.755, 0.764] | 0.070 [0.068, 0.072] | 0.0264 | 0.0071 |
| excl_reconnect_1h | 1 | 299917 | 143 | 0.05 | 0.762 [0.758, 0.767] | 0.069 [0.067, 0.072] | 0.0260 | 0.0068 |
| excl_reconnect_4h | 4 | 299917 | 143 | 0.05 | 0.762 [0.758, 0.767] | 0.069 [0.067, 0.072] | 0.0260 | 0.0068 |
| excl_reconnect_12h | 12 | 299917 | 143 | 0.05 | 0.762 [0.758, 0.767] | 0.069 [0.067, 0.072] | 0.0260 | 0.0068 |
| excl_reconnect_24h | 24 | 299917 | 143 | 0.05 | 0.762 [0.758, 0.767] | 0.069 [0.067, 0.072] | 0.0260 | 0.0068 |

## Supplementary Table S3

Supplementary Table S3: Hyperparameter Tuning Results

Comparison of default versus grid-searched hyperparameters for all algorithm-phase-feature combinations, evaluated on the T3b holdout set. Grid search used nested 3-fold inner CV within each training phase. Random Forest parameters: three mtry values per configuration (spanning 2-8, finalized from each feature set's predictor count) and min_n (5, 10, 20, 50). CatBoost parameters: depth (4, 6, 8, 10) and learning_rate (0.01, 0.05, 0.1). Deltas are within ±0.015 for all with_agents configurations (the recommended CatBoost T2/with_agents configuration is 0.767 default vs 0.759 tuned); the one larger change is CatBoost T1/base (−0.059), a configuration already below chance on T3b. Algorithm performance differences persist after tuning, reflecting architecture rather than parameter suitability.

| Algorithm | Phase | Features | Default ROC-AUC | Tuned ROC-AUC | Delta |
| --- | --- | --- | --- | --- | --- |
| CatBoost | T1 | base | 0.375 | 0.316 | -0.059 |
| CatBoost | T1 | with_agents | 0.715 | 0.723 | +0.008 |
| CatBoost | T2 | base | 0.599 | 0.608 | +0.008 |
| CatBoost | T2 | with_agents | 0.767 | 0.759 | -0.008 |
| CatBoost | T3a | base | 0.599 | 0.609 | +0.010 |
| CatBoost | T3a | with_agents | 0.760 | 0.774 | +0.015 |
| RF | T1 | base | 0.321 | 0.324 | +0.004 |
| RF | T1 | with_agents | 0.549 | 0.545 | -0.005 |
| RF | T2 | base | 0.608 | 0.598 | -0.009 |
| RF | T2 | with_agents | 0.715 | 0.725 | +0.010 |
| RF | T3a | base | 0.599 | 0.601 | +0.002 |
| RF | T3a | with_agents | 0.746 | 0.754 | +0.008 |

## Supplementary Table S4

Supplementary Table S4: Blocked Temporal Cross-Validation Versus Standard 5-Fold CV

Comparison of expanding-window blocked temporal CV against standard 5-fold CV for all 12 algorithm-phase-feature configurations (no imbalance correction variant). Blocked CV uses monthly expanding windows within each ~4-month training phase. Blocked CV consistently produces lower ROC-AUC estimates (mean delta: −0.079), confirming temporal autocorrelation inflates standard CV estimates. Model ranking is preserved under both CV schemes; T1-trained with_agents models remain above 0.80 AUC under blocked CV, while T2- and T3a-trained with_agents models fall to 0.656-0.663, widening the blocked-vs-standard optimism gap for the recommended configuration to 0.115.

| Algorithm | Phase | Features | Blocked CV AUC | Standard 5-Fold AUC | Delta |
| --- | --- | --- | --- | --- | --- |
| CatBoost | T1 | base | 0.771 | 0.797 | -0.026 |
| CatBoost | T1 | with_agents | 0.811 | 0.833 | -0.022 |
| CatBoost | T2 | base | 0.567 | 0.661 | -0.094 |
| CatBoost | T2 | with_agents | 0.663 | 0.778 | -0.115 |
| CatBoost | T3a | base | 0.542 | 0.671 | -0.129 |
| CatBoost | T3a | with_agents | 0.659 | 0.757 | -0.097 |
| RF | T1 | base | 0.760 | 0.788 | -0.028 |
| RF | T1 | with_agents | 0.819 | 0.825 | -0.006 |
| RF | T2 | base | 0.565 | 0.652 | -0.087 |
| RF | T2 | with_agents | 0.656 | 0.784 | -0.128 |
| RF | T3a | base | 0.540 | 0.668 | -0.128 |
| RF | T3a | with_agents | 0.660 | 0.750 | -0.091 |

## Supplementary Table S5

Supplementary Table S5: Baseline Model Comparison

Performance of four baseline models on the T3b holdout set, compared against the best ML model configuration. Logistic Regression = L2-regularized logistic regression with the same with_agents feature set; Erlang-A = queueing-theoretic model; Temporal Heuristic = hour-of-day abandonment rate lookup; Prevalence = predicting the marginal abandonment rate for all calls. ML uplift shows the absolute ROC-AUC improvement of the best ML model (CatBoost T2/with_agents/none, ROC-AUC = 0.767) over each baseline.

| Baseline Model | Best ROC-AUC | Best PR-AUC | Mean ROC-AUC | N Configs | ML Uplift (ROC-AUC) | ML Uplift (%) |
| --- | --- | --- | --- | --- | --- | --- |
| Erlang-A | 0.561 | 0.031 | 0.561 | 3 | 0.206 | 36.7% |
| Temporal Heuristic | 0.562 | 0.034 | 0.548 | 3 | 0.205 | 36.4% |
| Logistic Regression | 0.736 | 0.075 | 0.591 | 6 | 0.031 | 4.2% |
| Prevalence | 0.500 | 0.0276 | 0.500 | 6 | 0.267 | 53.4% |

## Supplementary Table S6

Supplementary Table S6: PR-AUC Lift Over Prevalence Baseline. PR-AUC lift for all 36 model configurations on the T3b holdout set, computed as model PR-AUC divided by the T3b prevalence baseline (0.0276). With_agents configurations trained on T2 or T3a achieve 2.07×-2.58× lift (2.47× for the recommended CatBoost T2/with_agents/none); T1-trained with_agents configurations degrade to 0.95×-1.90×, with RF T1/with_agents/smote falling below the prevalence baseline. Base-feature configurations cluster between 0.66× and 1.40×, demonstrating that knowability-gated operational features still substantially improve performance for models trained after the operational transition.

| Algorithm | Training Phase | Feature Set | Variant | PR-AUC | PR-AUC Lift |
| --- | --- | --- | --- | --- | --- |
| CatBoost | T2 | with_agents | class_weights | 0.071 | 2.58× |
| CatBoost | T3a | with_agents | none | 0.070 | 2.53× |
| RF | T3a | with_agents | smote | 0.069 | 2.48× |
| CatBoost | T2 | with_agents | smote | 0.068 | 2.48× |
| RF | T3a | with_agents | class_weights | 0.068 | 2.47× |
| RF | T3a | with_agents | none | 0.068 | 2.47× |
| CatBoost | T2 | with_agents | none | 0.068 | 2.47× |
| CatBoost | T3a | with_agents | smote | 0.068 | 2.44× |
| CatBoost | T3a | with_agents | class_weights | 0.066 | 2.39× |
| RF | T2 | with_agents | smote | 0.061 | 2.19× |
| RF | T2 | with_agents | none | 0.058 | 2.08× |
| RF | T2 | with_agents | class_weights | 0.057 | 2.07× |
| CatBoost | T1 | with_agents | class_weights | 0.052 | 1.90× |
| CatBoost | T1 | with_agents | none | 0.051 | 1.83× |
| CatBoost | T1 | with_agents | smote | 0.045 | 1.65× |
| RF | T2 | base | class_weights | 0.039 | 1.40× |
| RF | T2 | base | none | 0.039 | 1.40× |
| CatBoost | T2 | base | smote | 0.038 | 1.36× |
| RF | T3a | base | smote | 0.038 | 1.36× |
| RF | T2 | base | smote | 0.038 | 1.36× |
| CatBoost | T3a | base | none | 0.037 | 1.35× |
| RF | T3a | base | class_weights | 0.037 | 1.35× |
| RF | T3a | base | none | 0.037 | 1.34× |
| CatBoost | T2 | base | class_weights | 0.037 | 1.34× |
| CatBoost | T3a | base | class_weights | 0.037 | 1.33× |
| CatBoost | T3a | base | smote | 0.037 | 1.33× |
| CatBoost | T2 | base | none | 0.037 | 1.33× |
| RF | T1 | with_agents | class_weights | 0.030 | 1.09× |
| RF | T1 | with_agents | none | 0.030 | 1.09× |
| RF | T1 | with_agents | smote | 0.026 | 0.95× |
| CatBoost | T1 | base | class_weights | 0.021 | 0.77× |
| CatBoost | T1 | base | none | 0.020 | 0.72× |
| CatBoost | T1 | base | smote | 0.020 | 0.72× |
| RF | T1 | base | class_weights | 0.018 | 0.67× |
| RF | T1 | base | none | 0.018 | 0.67× |
| RF | T1 | base | smote | 0.018 | 0.66× |

## Supplementary Table S7

Supplementary Table S7: Feature Set Comparison on T3b Holdout

Performance of all 36 model configurations on the T3b holdout set, organized by algorithm, training phase, feature set, and imbalance handling variant. Adding agent staffing features (with_agents) consistently improves ROC-AUC by 0.11-0.34 over the base temporal-only features, regardless of algorithm or training phase.

| Algorithm | Training Phase | Feature Set | Variant | ROC-AUC | PR-AUC | Brier | ECE |
| --- | --- | --- | --- | --- | --- | --- | --- |
| RF | T1 | base | none | 0.321 | 0.018 | 0.040 | 0.080 |
| RF | T1 | base | class_weights | 0.322 | 0.018 | 0.040 | 0.080 |
| RF | T1 | base | smote | 0.308 | 0.018 | 0.065 | 0.132 |
| RF | T1 | with_agents | none | 0.549 | 0.030 | 0.029 | 0.026 |
| RF | T1 | with_agents | class_weights | 0.552 | 0.030 | 0.029 | 0.025 |
| RF | T1 | with_agents | smote | 0.499 | 0.026 | 0.033 | 0.047 |
| RF | T2 | base | none | 0.608 | 0.039 | 0.028 | 0.027 |
| RF | T2 | base | class_weights | 0.608 | 0.039 | 0.028 | 0.027 |
| RF | T2 | base | smote | 0.602 | 0.038 | 0.058 | 0.157 |
| RF | T2 | with_agents | none | 0.715 | 0.058 | 0.027 | 0.017 |
| RF | T2 | with_agents | class_weights | 0.714 | 0.057 | 0.027 | 0.017 |
| RF | T2 | with_agents | smote | 0.724 | 0.061 | 0.030 | 0.040 |
| RF | T3a | base | none | 0.599 | 0.037 | 0.027 | 0.007 |
| RF | T3a | base | class_weights | 0.599 | 0.037 | 0.027 | 0.007 |
| RF | T3a | base | smote | 0.609 | 0.038 | 0.058 | 0.156 |
| RF | T3a | with_agents | none | 0.746 | 0.068 | 0.027 | 0.007 |
| RF | T3a | with_agents | class_weights | 0.744 | 0.068 | 0.027 | 0.007 |
| RF | T3a | with_agents | smote | 0.749 | 0.069 | 0.029 | 0.033 |
| CatBoost | T1 | base | none | 0.375 | 0.020 | 0.050 | 0.072 |
| CatBoost | T1 | base | class_weights | 0.412 | 0.021 | 0.145 | 0.226 |
| CatBoost | T1 | base | smote | 0.368 | 0.020 | 0.067 | 0.110 |
| CatBoost | T1 | with_agents | none | 0.715 | 0.051 | 0.027 | 0.013 |
| CatBoost | T1 | with_agents | class_weights | 0.708 | 0.052 | 0.032 | 0.038 |
| CatBoost | T1 | with_agents | smote | 0.691 | 0.045 | 0.029 | 0.022 |
| CatBoost | T2 | base | none | 0.599 | 0.037 | 0.030 | 0.030 |
| CatBoost | T2 | base | class_weights | 0.599 | 0.037 | 0.229 | 0.407 |
| CatBoost | T2 | base | smote | 0.600 | 0.038 | 0.060 | 0.153 |
| CatBoost | T2 | with_agents | none | 0.767 | 0.068 | 0.027 | 0.006 |
| CatBoost | T2 | with_agents | class_weights | 0.761 | 0.071 | 0.084 | 0.171 |
| CatBoost | T2 | with_agents | smote | 0.763 | 0.068 | 0.027 | 0.011 |
| CatBoost | T3a | base | none | 0.599 | 0.037 | 0.027 | 0.012 |
| CatBoost | T3a | base | class_weights | 0.596 | 0.037 | 0.225 | 0.405 |
| CatBoost | T3a | base | smote | 0.594 | 0.037 | 0.059 | 0.149 |
| CatBoost | T3a | with_agents | none | 0.760 | 0.070 | 0.026 | 0.007 |
| CatBoost | T3a | with_agents | class_weights | 0.739 | 0.066 | 0.092 | 0.169 |
| CatBoost | T3a | with_agents | smote | 0.752 | 0.068 | 0.027 | 0.010 |

## Supplementary Table S8

Supplementary Table S8: Temporal Generalization Analysis. Performance change for all model configurations between within-phase in-sample evaluation (“In-sample AUC,” computed by training and evaluating each model on its own training phase, i.e., the diagonal cells of Figure 4 Panel A) and the prospective T3b holdout (“T3b AUC”). In-sample values are NOT the five-fold cross-validation values reported in Table 2 of the main manuscript; the two quantities differ by construction and are not directly comparable (e.g., CatBoost T2/with_agents/none: Table 2 CV AUC = 0.778; within-phase in-sample AUC here = 0.849). All configurations decline from within-phase in-sample fit to the prospective holdout, as expected when comparing optimistic same-phase evaluation against prospective generalization. CatBoost models with agent features decline by 9%-15% (T2-trained) and 13%-23% (T3a-trained); Random Forest models with agent features decline more (20%-28%), reflecting their higher within-phase fit (0.92-0.99). Models trained on T1, and base-feature models, show the largest declines (up to approximately 62%), consistent with operational drift from the earliest phase and the limited signal in temporal-only features.

| Algorithm | Train Phase | Feature Set | Variant | In-sample AUC | T3b AUC | AUC Drop | % Drop |
| --- | --- | --- | --- | --- | --- | --- | --- |
| RF | T1 | with_agents | none | 0.972 | 0.549 | 0.423 | 43.5% |
| RF | T1 | with_agents | class_weights | 0.972 | 0.552 | 0.420 | 43.2% |
| RF | T1 | with_agents | smote | 0.921 | 0.499 | 0.422 | 45.8% |
| RF | T1 | base | none | 0.791 | 0.321 | 0.470 | 59.4% |
| RF | T1 | base | class_weights | 0.791 | 0.322 | 0.469 | 59.2% |
| RF | T1 | base | smote | 0.802 | 0.308 | 0.493 | 61.5% |
| RF | T2 | with_agents | none | 0.990 | 0.715 | 0.275 | 27.7% |
| RF | T2 | with_agents | class_weights | 0.990 | 0.714 | 0.276 | 27.9% |
| RF | T2 | with_agents | smote | 0.923 | 0.724 | 0.199 | 21.6% |
| RF | T2 | base | none | 0.662 | 0.608 | 0.054 | 8.2% |
| RF | T2 | base | class_weights | 0.661 | 0.608 | 0.053 | 8.1% |
| RF | T2 | base | smote | 0.675 | 0.602 | 0.073 | 10.8% |
| RF | T3a | with_agents | none | 0.995 | 0.746 | 0.250 | 25.1% |
| RF | T3a | with_agents | class_weights | 0.995 | 0.744 | 0.251 | 25.2% |
| RF | T3a | with_agents | smote | 0.942 | 0.749 | 0.194 | 20.5% |
| RF | T3a | base | none | 0.685 | 0.599 | 0.087 | 12.6% |
| RF | T3a | base | class_weights | 0.685 | 0.599 | 0.086 | 12.5% |
| RF | T3a | base | smote | 0.692 | 0.609 | 0.082 | 11.9% |
| CatBoost | T1 | with_agents | none | 0.880 | 0.715 | 0.166 | 18.8% |
| CatBoost | T1 | with_agents | class_weights | 0.894 | 0.708 | 0.187 | 20.9% |
| CatBoost | T1 | with_agents | smote | 0.875 | 0.691 | 0.184 | 21.0% |
| CatBoost | T1 | base | none | 0.803 | 0.375 | 0.429 | 53.3% |
| CatBoost | T1 | base | class_weights | 0.804 | 0.412 | 0.391 | 48.7% |
| CatBoost | T1 | base | smote | 0.803 | 0.368 | 0.435 | 54.1% |
| CatBoost | T2 | with_agents | none | 0.849 | 0.767 | 0.082 | 9.7% |
| CatBoost | T2 | with_agents | class_weights | 0.891 | 0.761 | 0.130 | 14.6% |
| CatBoost | T2 | with_agents | smote | 0.840 | 0.763 | 0.076 | 9.1% |
| CatBoost | T2 | base | none | 0.678 | 0.599 | 0.078 | 11.6% |
| CatBoost | T2 | base | class_weights | 0.678 | 0.599 | 0.079 | 11.7% |
| CatBoost | T2 | base | smote | 0.677 | 0.600 | 0.077 | 11.3% |
| CatBoost | T3a | with_agents | none | 0.873 | 0.760 | 0.114 | 13.0% |
| CatBoost | T3a | with_agents | class_weights | 0.956 | 0.739 | 0.217 | 22.7% |
| CatBoost | T3a | with_agents | smote | 0.865 | 0.752 | 0.114 | 13.1% |
| CatBoost | T3a | base | none | 0.702 | 0.599 | 0.103 | 14.6% |
| CatBoost | T3a | base | class_weights | 0.703 | 0.596 | 0.106 | 15.1% |
| CatBoost | T3a | base | smote | 0.697 | 0.594 | 0.102 | 14.7% |

## Supplementary Table S9

Supplementary Table S9: Imbalance Handling Method Comparison on T3b Holdout

Performance comparison of three imbalance handling strategies (none, class weights, SMOTE) averaged across training phases, stratified by algorithm and feature set. No single imbalance method consistently dominates; the unweighted ('none') variant achieves competitive or best ROC-AUC in most configurations, while SMOTE provides marginal benefit and class weights are competitive but can degrade calibration (ECE).

| Algorithm | Feature Set | Variant | Mean ROC-AUC | Mean PR-AUC | Mean Brier | Mean ECE |
| --- | --- | --- | --- | --- | --- | --- |
| RF | base | none | 0.509 | 0.031 | 0.032 | 0.038 |
| RF | base | class_weights | 0.510 | 0.031 | 0.032 | 0.038 |
| RF | base | smote | 0.507 | 0.031 | 0.060 | 0.148 |
| RF | with_agents | none | 0.670 | 0.052 | 0.028 | 0.017 |
| RF | with_agents | class_weights | 0.670 | 0.052 | 0.028 | 0.017 |
| RF | with_agents | smote | 0.657 | 0.052 | 0.031 | 0.040 |
| CatBoost | base | none | 0.525 | 0.031 | 0.036 | 0.038 |
| CatBoost | base | class_weights | 0.536 | 0.032 | 0.200 | 0.346 |
| CatBoost | base | smote | 0.521 | 0.031 | 0.062 | 0.137 |
| CatBoost | with_agents | none | 0.747 | 0.063 | 0.027 | 0.009 |
| CatBoost | with_agents | class_weights | 0.736 | 0.063 | 0.069 | 0.126 |
| CatBoost | with_agents | smote | 0.735 | 0.061 | 0.028 | 0.014 |

## Supplementary Table S10

Supplementary Table S10: Algorithm Comparison Summary on T3b Holdout

Aggregate performance comparison between Random Forest and CatBoost across all 18 configurations per algorithm on the T3b holdout set. CatBoost achieves a higher mean ROC-AUC (0.633 vs 0.587) and higher maximum ROC-AUC (0.767 vs 0.749), with a narrower standard deviation, suggesting more stable performance across training phases and feature configurations.

| Algorithm | N Models | Mean ROC-AUC | SD ROC-AUC | Median ROC-AUC | Min ROC-AUC | Max ROC-AUC | Mean PR-AUC | Mean Brier | Mean ECE |
| --- | --- | --- | --- | --- | --- | --- | --- | --- | --- |
| RF | 18 | 0.587 | 0.145 | 0.605 | 0.308 | 0.749 | 0.042 | 0.035 | 0.050 |
| CatBoost | 18 | 0.633 | 0.133 | 0.645 | 0.368 | 0.767 | 0.047 | 0.070 | 0.112 |

## Supplementary Table S11

Supplementary Table S11: Complete Model Leaderboard on T3b Holdout

All 36 model configurations ranked by T3b holdout ROC-AUC with 95% confidence intervals, PR-AUC, Brier score, and ECE. The top 15 models all use the with_agents feature set (the first base-feature configuration appears at rank 16), confirming that agent staffing features are the primary driver of discrimination performance. RF T1/with_agents configurations fall to ranks 28-30 (ROC-AUC 0.499-0.552), below all T2- and T3a-trained base models. Base-feature models cluster near 0.60 ROC-AUC regardless of algorithm, while T1-trained base models show substantial degradation (<0.42) due to temporal drift.

| Rank | Algorithm | Train Phase | Feature Set | Variant | ROC-AUC | 95% CI | PR-AUC | Brier | ECE |
| --- | --- | --- | --- | --- | --- | --- | --- | --- | --- |
| 1 | CatBoost | T2 | with_agents | none | 0.767 | (0.762-0.771) | 0.068 | 0.027 | 0.006 |
| 2 | CatBoost | T2 | with_agents | smote | 0.763 | (0.759-0.768) | 0.068 | 0.027 | 0.011 |
| 3 | CatBoost | T2 | with_agents | class_weights | 0.761 | (0.756-0.765) | 0.071 | 0.084 | 0.171 |
| 4 | CatBoost | T3a | with_agents | none | 0.760 | (0.755-0.764) | 0.070 | 0.026 | 0.007 |
| 5 | CatBoost | T3a | with_agents | smote | 0.752 | (0.747-0.756) | 0.068 | 0.027 | 0.010 |
| 6 | RF | T3a | with_agents | smote | 0.749 | (0.744-0.754) | 0.069 | 0.029 | 0.033 |
| 7 | RF | T3a | with_agents | none | 0.746 | (0.741-0.751) | 0.068 | 0.027 | 0.007 |
| 8 | RF | T3a | with_agents | class_weights | 0.744 | (0.739-0.749) | 0.068 | 0.027 | 0.007 |
| 9 | CatBoost | T3a | with_agents | class_weights | 0.739 | (0.734-0.744) | 0.066 | 0.092 | 0.169 |
| 10 | RF | T2 | with_agents | smote | 0.724 | (0.719-0.730) | 0.061 | 0.030 | 0.040 |
| 11 | RF | T2 | with_agents | none | 0.715 | (0.710-0.721) | 0.058 | 0.027 | 0.017 |
| 12 | CatBoost | T1 | with_agents | none | 0.715 | (0.710-0.719) | 0.051 | 0.027 | 0.013 |
| 13 | RF | T2 | with_agents | class_weights | 0.714 | (0.709-0.720) | 0.057 | 0.027 | 0.017 |
| 14 | CatBoost | T1 | with_agents | class_weights | 0.708 | (0.703-0.712) | 0.052 | 0.032 | 0.038 |
| 15 | CatBoost | T1 | with_agents | smote | 0.691 | (0.686-0.696) | 0.045 | 0.029 | 0.022 |
| 16 | RF | T3a | base | smote | 0.609 | (0.603-0.615) | 0.038 | 0.058 | 0.156 |
| 17 | RF | T2 | base | class_weights | 0.608 | (0.602-0.614) | 0.039 | 0.028 | 0.027 |
| 18 | RF | T2 | base | none | 0.608 | (0.602-0.613) | 0.039 | 0.028 | 0.027 |
| 19 | RF | T2 | base | smote | 0.602 | (0.596-0.608) | 0.038 | 0.058 | 0.157 |
| 20 | CatBoost | T2 | base | smote | 0.600 | (0.594-0.606) | 0.038 | 0.060 | 0.153 |
| 21 | CatBoost | T2 | base | none | 0.599 | (0.594-0.605) | 0.037 | 0.030 | 0.030 |
| 22 | RF | T3a | base | class_weights | 0.599 | (0.593-0.605) | 0.037 | 0.027 | 0.007 |
| 23 | CatBoost | T3a | base | none | 0.599 | (0.593-0.605) | 0.037 | 0.027 | 0.012 |
| 24 | RF | T3a | base | none | 0.599 | (0.593-0.605) | 0.037 | 0.027 | 0.007 |
| 25 | CatBoost | T2 | base | class_weights | 0.599 | (0.593-0.605) | 0.037 | 0.229 | 0.407 |
| 26 | CatBoost | T3a | base | class_weights | 0.596 | (0.590-0.602) | 0.037 | 0.225 | 0.405 |
| 27 | CatBoost | T3a | base | smote | 0.594 | (0.588-0.600) | 0.037 | 0.059 | 0.149 |
| 28 | RF | T1 | with_agents | class_weights | 0.552 | (0.546-0.558) | 0.030 | 0.029 | 0.025 |
| 29 | RF | T1 | with_agents | none | 0.549 | (0.543-0.555) | 0.030 | 0.029 | 0.026 |
| 30 | RF | T1 | with_agents | smote | 0.499 | (0.493-0.505) | 0.026 | 0.033 | 0.047 |
| 31 | CatBoost | T1 | base | class_weights | 0.412 | (0.407-0.417) | 0.021 | 0.145 | 0.226 |
| 32 | CatBoost | T1 | base | none | 0.375 | (0.370-0.380) | 0.020 | 0.050 | 0.072 |
| 33 | CatBoost | T1 | base | smote | 0.368 | (0.363-0.373) | 0.020 | 0.067 | 0.110 |
| 34 | RF | T1 | base | class_weights | 0.322 | (0.317-0.328) | 0.018 | 0.040 | 0.080 |
| 35 | RF | T1 | base | none | 0.321 | (0.315-0.326) | 0.018 | 0.040 | 0.080 |
| 36 | RF | T1 | base | smote | 0.308 | (0.303-0.313) | 0.018 | 0.065 | 0.132 |
